# Arabidopsis HY5 enhances jasmonate responses by directly suppressing the expression of the *JAZ1* gene in a FIN219/JAR1-dependent manner

**DOI:** 10.3389/fpls.2026.1891433

**Published:** 2026-08-03

**Authors:** Yen-Ho Chen, Meng-Chun Lin, Li-Lin Liao, Kai-Chun Peng, Chia-Cheng Yen, Ang-Hsin Liu, Jhy-Gong Wang, Hsu-Liang Hsieh

**Affiliations:** 1 Institute of Plant Biology, College of Life Science, National Taiwan University, Taipei, Taiwan; 2 Institute of Plant and Microbial Biology, Academia Sinica, Taipei, Taiwan; 3 Department of Agronomy, National Chiayi University, Chiayi, Taiwan; 4 Department of Life Science, College of Life Science, National Taiwan University, Taipei, Taiwan; 5 Master Program in Global Agriculture Technology and Genomic Science, National Taiwan University, Taipei, Taiwan

**Keywords:** bZIP transcription factor, FIN219/JAR1, HY5, jasmonates, photomorphogenesis, seedling development

## Abstract

Light is a key environmental signal that regulates plant growth and development. ELONGATED HYPOCOTYL 5 (HY5), a bZIP transcription factor, is a central positive regulator of photomorphogenesis in *Arabidopsis thaliana*, integrating light and hormone signaling pathways. However, the molecular role of HY5 in jasmonate (JA) signaling remains unclear. Here, we show that methyl jasmonate (MeJA) substantially induces HY5 protein accumulation in a FIN219/JAR1-dependent manner. Genetic evidence indicates that HY5 and FIN219 act synergistically to inhibit hypocotyl growth and coordinate defense-related transcriptional networks. Loss of HY5 function results in hypersensitivity to MeJA-mediated inhibition of hypocotyl elongation, and transcriptomic analyses reveal disturbances in numerous light- and JA-responsive genes. HY5 represses *JAZ1* expression by binding to its G-box-containing promoter and physically interacts with JAZ1, suggesting both transcriptional and post-translational regulation. Furthermore, HY5 and MYC2 compete for binding at the *JAZ1* promoter, modulating JA responses under far-red light. Together, our findings uncover a regulatory mechanism in which HY5 fine-tunes JA signaling and seedling development by integrating light and hormone cues. This work provides new insights into HY5-mediated crosstalk and identifies potential targets to improve stress resilience in crops.

## Highlight

- HY5 is dramatically induced by jasmonates and enhances light signaling by suppressing the repressor JAZ1 function in jasmonate signaling through direct interaction, thereby regulating the photomorphogenic development of Arabidopsis seedlings.

## Introduction

Plants continuously adjust their growth and development in response to dynamic environmental cues, notably light and phytohormones. In *Arabidopsis thaliana*, light signals are perceived by a broad spectrum of photoreceptors, including phytochromes (phys) for red light (R)/far-red light (FR) and cryptochromes (crys) for blue light (Huq et al., 2024). Light signaling pathways converge on ELONGATED HYPOCOTYL 5 (HY5), a basic leucine-zipper (bZIP) transcription factor (Oyama et al., 1997). HY5 interacts with the ubiquitin E3 ligase CONSTITUTIVE PHOTOMORPHOGENIC 1 (COP1), which targets HY5 for 26S proteasome-mediated degradation in darkness, resulting in elongated hypocotyls and closed cotyledons. Upon light exposure, COP1 is exported to the cytosol and can no longer mediate HY5 degradation, leading to rapid expression of light-responsive genes that drive photomorphogenic growth of seedlings (Ang et al., 1998).

Beyond light, HY5 is involved in phytohormone signaling, including jasmonate (Hsieh and Okamoto, 2014; Campos et al., 2016), auxin (Cluis et al., 2004), gibberellins (Weller et al., 2009; Xiong et al., 2023), abscisic acid (ABA) signaling (Bhagat et al., 2021), and various stress responses (Kindgren et al., 2012; Nawkar et al., 2017; Zhang et al., 2011). Jasmonic acid (JA) and its derivatives, collectively referred to as jasmonates (JAs), are synthesized from α-linolenic acid in the chloroplast membrane and are involved in diverse developmental events and defense responses (Kazan and Manners, 2008). Under mechanical wounding or pathogen attack, JA is rapidly synthesized and conjugated with the amino acid isoleucine (Ile) by the JA-conjugating enzyme FAR-RED INSENSITIVE 219 (FIN219)/JASMONIC ACID RESISTANT 1 (JAR1), forming the active form JA-Ile (Wang et al., 2011; Staswick and Tiryaki, 2004). The receptor CORONATINE INSENSITIVE 1 (COI1) is the F-box protein of a SKP-CULLIN-F-BOX (SCF) type ubiquitin E3 ligase that perceives JA-Ile, triggering degradation of the JA signaling repressors JASMONIC ACID ZIM-DOMAIN (JAZs) (Chini et al., 2007; Thines et al., 2007). The JAZ proteins interact with MYC2, a central basic helix-loop-helix (bHLH) transcriptional activator of JA-responsive genes, thereby modulating the expression of defense-related genes against herbivores and fungal pathogens (Boter et al., 2004; Lorenzo et al., 2004; Dombrecht et al., 2007; Browse, 2009). Additional bHLH transcription factors, such as MYC3 and MYC4, are also involved in JA signaling (Cheng et al., 2011; Fernández-Calvo et al., 2011; Niu et al., 2011). Arabidopsis MYC2, MYC3, and MYC4 function redundantly in seed storage protein accumulation and additively in wound-induced JA metabolism (Gao et al., 2016).

FIN219/JAR1 and HY5 are extragenic suppressors of COP1 (Ang and Deng, 1994; Hsieh et al., 2000). FIN219/JAR1 negatively regulates COP1 protein levels through direct interaction. Moreover, ectopic expression of FIN219 alters COP1 subcellular localization from the nucleus to the cytoplasm, even under dark conditions, resulting in a substantially shorter hypocotyl phenotype (Wang et al., 2011). Our recent results reveal that FIN219 interacts with multiple photoreceptors, including phyA, phyB, and cryptochrome 1, under FR, shade, and blue light, respectively, to regulate seedling development (Jiang et al., 2023; Peng et al., 2023; Chen et al., 2018), suggesting that FIN219 plays a pivotal role in integrating light and JA signaling pathways.

Previous studies showed that *HY5* and *HY1*, an HY5 enhancer involved in phytochrome chromophore biosynthesis, synergistically regulate light responses, including chlorophyll and anthocyanin accumulation, JA-mediated gene expression, and inhibition of root elongation (Prasad et al., 2012). Further reports revealed that red light-induced gene expression required MYC transcription factors. Moreover, MYCs could directly bind to the *HY5* promoter, leading to a photomorphogenic response (Ortigosa et al., 2020; Yi et al., 2020). To date, the role of HY5 in JA signaling has been understood primarily at the transcriptional level. However, how HY5 protein abundance is modulated by JA, and the molecular mechanisms underlying the requirement of FIN219 for COP1-mediated regulation of HY5 stability under light conditions, remain largely unclear. Previous studies have demonstrated crosstalk between phytochrome-mediated light signaling and JA pathways (Campos et al., 2016; Robson et al., 2010), yet the key points of integration between these signaling networks remain elusive. In this study, we report a physical interaction between HY5 and the JA signaling repressor JAZ1, which is strongly dependent on functional FIN219. These findings suggest that the HY5–JAZ1 association may represent a novel regulatory node that integrates light and JA signaling pathways in Arabidopsis.

## Materials and methods

### Plant materials

To clarify the genetic regulatory relationship between FIN219 and HY5 in the light and JA signaling pathways, we established a *hy5* null mutant in the Col-0 background (*hy5-ks50/Col-0*) by introgressing the *hy5-ks50* mutation from the Wassilewskija (Ws) ecotype into Col-0 through five rounds of backcrossing. We confirmed the absence of the *phyD* mutation, a single-nucleotide substitution in the *PHYD* gene present in Ws, as previously described (Aukerman et al., 1997). To test genetic interactions, we also generated the double mutants *hy5-ks50* (Col-0 background) *fin219–2* and *hy5-ks50 pGR219* (*pGR219*: *FIN219* inducible overexpression line). In addition, *hy5–1* in the *Landsberg erecta* (*Ler*) ecotype background was used in studies of anthocyanin accumulation, microarray assays, and gene expression. The *fin219–2* null mutant and *pGR219* were also in the Col-0 background, as described previously (Wang et al., 2011).

### Plant growth and phenotyping conditions

Seeds were sterilized and sown on germination medium (GM) as described previously (Wang et al., 2011). Seeds were stratified for two days at 4 °C in darkness, then exposed to 16 h of continuous white light (cWL, 70-80 μmol m^-2^s^-1^) at 22 °C to synchronize germination. Synchronized seeds were assigned to the following treatments. The expected growth condition was 22 °C under long-day light (16 h light/8 h dark) to complete the Arabidopsis life cycle. Short-day light (8 h light/16 h dark) was used to arrest seedlings in the vegetative stage for rosette leaf collection for protoplast preparation. In experiments on MeJA-mediated growth inhibition, Arabidopsis materials were grown on GM plates with or without 50 μM MeJA at 22 °C under the following light conditions: continuous white light (cWL, 70 μmol m^-2^s^-1^), far-red light (cFR, 1.5 μmol m^-2^s^-1^), red light (cRL, 5.0 μmol m^-2^s^-1^), blue light (cBL, 2.2 μmol m^-2^s^-1^), and darkness (Dark, n.d. quantum). The 4-day-old and 10-day-old Arabidopsis seedlings were used to investigate MeJA-mediated inhibition of hypocotyl and primary root lengths, respectively. Plants were grown vertically on the medium surface to promote root growth for root phenotyping. We captured images and measured the lengths of hypocotyls and primary roots using ImageJ (Schneider et al., 2012). The MeJA-mediated inhibition percentage was calculated using the formula [(L^ETOH^-L^MeJA^)/L^ETOH^]*100%, where L denotes the measured length of hypocotyls or primary roots.

### Total protein extraction and Western blotting assays

Total proteins were extracted from frozen plants after pulverization in liquid nitrogen. We then mixed the powdered samples with protein extraction buffer (50 mM Tris, pH 7.5; 150 mM NaCl; 10 mM MgCl2; 0.1% NP-40; 1 mM PMSF; 1X Protease inhibitor cocktail) and centrifuged at 15,000 × g for 15 min at 4 °C to collect the pellet. The Bradford Protein Assay kit (Bio-Rad) was used to determine total protein concentration. Equal amounts of total protein were separated by 15% SDS-PAGE and transferred to a PVDF membrane (Millipore). As described previously, the 26S proteasome subunit RPN8 was used as a loading control (Chen et al., 2018). After immunohybridization, ECL solution (iNtRON Co.) was applied to the membrane and the membrane was exposed to a LAS-3000 luminescent image analyzer (FujiFilm Co.). The primary antibodies used were polyclonal anti-FIN219 (Chen et al., 2007) against the N-terminal 300 amino acids of FIN219; anti-COP1 and anti-HY5 were obtained from Dr. Xing-Wang Deng; and anti-RPN8 was obtained from Dr. Hongyong Fu (Academia Sinica). The secondary antibody was commercial anti-rabbit-IgG (SantaCruz Co.). The signal was detected using an HRP substrate (EMD Millipore, ImmobilonTM) and imaged with a UVP AutoChemiTM Image System.

### Microarray sample preparation and bioinformatics assays

We harvested four-day-old *Ler* and *hy5–1* mutant seedlings grown on GM plates with or without 50 μM MeJA under cFR light for total RNA extraction. Isolated total RNA was assessed for quality and used for library preparation. Libraries were analyzed on the Affymetrix ATH1 genome array. Raw data were normalized using GeneSpring GX. We considered genes with fold changes greater than 2 or less than 0.5 in the *hy5–1* mutant relative to *Ler*, under FR light or darkness, with or without MeJA treatment, as differentially expressed. Functional characterization of differentially expressed genes was conducted using Gene Ontology (GO) and Kyoto Encyclopedia of Genes and Genomes (KEGG) with DAVID V6.7 (Huang et al., 2009). GO terms and KEGG pathways with p < 0.01 in Fisher’s exact test were considered enriched, using all expressed genes in our microarray analyses as the background reference. Expression profiles from this study are included in the supplemental data (**Supplementary Figure 4**), and the gene expression and raw data have been submitted to Gene Expression Omnibus (GEO) under accession number GSE336873.

### Yeast two-hybrid assay

The coding sequences of *COP1*, *HY5*, and *JAZs*, along with various deleted forms of *JAZ1* and *HY5*, were cloned into the DNA-binding domain vector pEG202 as bait and the activation domain vector pJG4–5 as prey, respectively. Primers used in the cloning process are listed in **Supplementary Table 3**. Constructs were transformed into yeast strain EGY48 using lithium acetate (Chen et al., 1992). Each transformation used 1 μg of each plasmid construct. Transformed colonies were selected on SD -Ura/-His/-Trp medium at 30 °C for 3 days. Colonies that grew on the medium were picked and grown on SD -Ura/-His/-Trp or SD -Ura/-His/-Trp/-Leu plates containing galactose, raffinose, and X-gal, then incubated at 30 °C for coloration.

### Bimolecular fluorescence complementation assay

We cloned the *nYFP-JAZ1* and *HY5-cYFP* sequences into the “2in1” system (Grefen and Blatt, 2012). Arabidopsis mesophyll isolation and transfection were performed as previously described (Yoo et al., 2007; Wu et al., 2009). The pBiFCt-2in1-NC-JM, which carries *nYFP-JAZ1* and *MYC2-cYFP*, served as a positive control. The pBiFCt-2in1-NC-JE, containing *nYFP-JAZ1* and *cYFP-*only, served as a negative control. For BiFC, 5 μg of each “2in1” plasmid was introduced into protoplasts (5 × 10^4^ cells per reaction) and the protoplasts were cultured under cWL for 16 hrs. We acquired images on a Zeiss LSM 780 Confocal Microscope, using standardized excitation intensities and photomultiplier gains, with the following excitation wavelength/emission bandwidth/dichroic filter settings: 488 nm/BP505-530/HFT488/543 (GFP) and 543 nm/BP560–615/HFT488/543 (RFP). The chlorophyll channel captured chlorophyll autofluorescence, the DAPI channel labeled the nucleus by 4,6-diamidino-2-phenylindole (DAPI) staining, and the merged channel combined YFP, RFP, DAPI, chlorophyll, and bright channels.

### Luciferase complementation imaging assays

The luciferase complementation imaging (LCI) assay was performed as described previously (Wang et al., 2021). The constructs *pGW-JAZ1-NLuc*, *pGW-HY5-CLuc*, *pGW-FIN219-NLuc*, *pGW-COP1-CLuc*, *pEG100-GFP*, and *pEG100-MYC2-GFP* were transformed into *Agrobacterium tumefaciens* strain GV3101 and co-infiltrated into *Nicotiana benthamiana* leaves. After a 24-hr incubation in darkness at 22 °C, followed by 24 hrs of continuous white light treatment, the leaves were harvested. A 1 mM D-luciferin (Abcam, ab143655) solution was injected into the tobacco leaves. Luciferase signals were captured with the iBright CL750 Imaging System (Invitrogen^™^). Quantitative analysis was performed using ImageJ software.

### Semi-co-immunoprecipitation assays

The recombinant protein His-JAZ1 was prepared in the *E. coli* expression system and purified using Ni-NTA resins. We extracted plant proteins from wild-type Col-0, the *hy5–1* mutant, and the 35S promoter-driven *HY5* overexpression line (*HY5 OE*) grown in FR light for 3 days, using extraction buffer (50 mM Tris-HCl, pH 7.5; 150 mM NaCl; 0.1% NP-40; 1X Protease Inhibitor Cocktail). Then, 1 μg of His-JAZ1 recombinant protein mixed with Ni-NTA resins was incubated with 1 mg of plant extract at 25 °C with shaking at 20 rpm for 30 min. After centrifugation at 100xg for 30 seconds, the supernatant was removed, and the pellets were washed three times with His-tag washing buffer and then subjected to Western blot analysis using polyclonal antibodies against HY5 and the His tag, respectively, as described previously (Wang et al., 2011).

### Electrophoretic mobility shift assay

**Figure 1:**
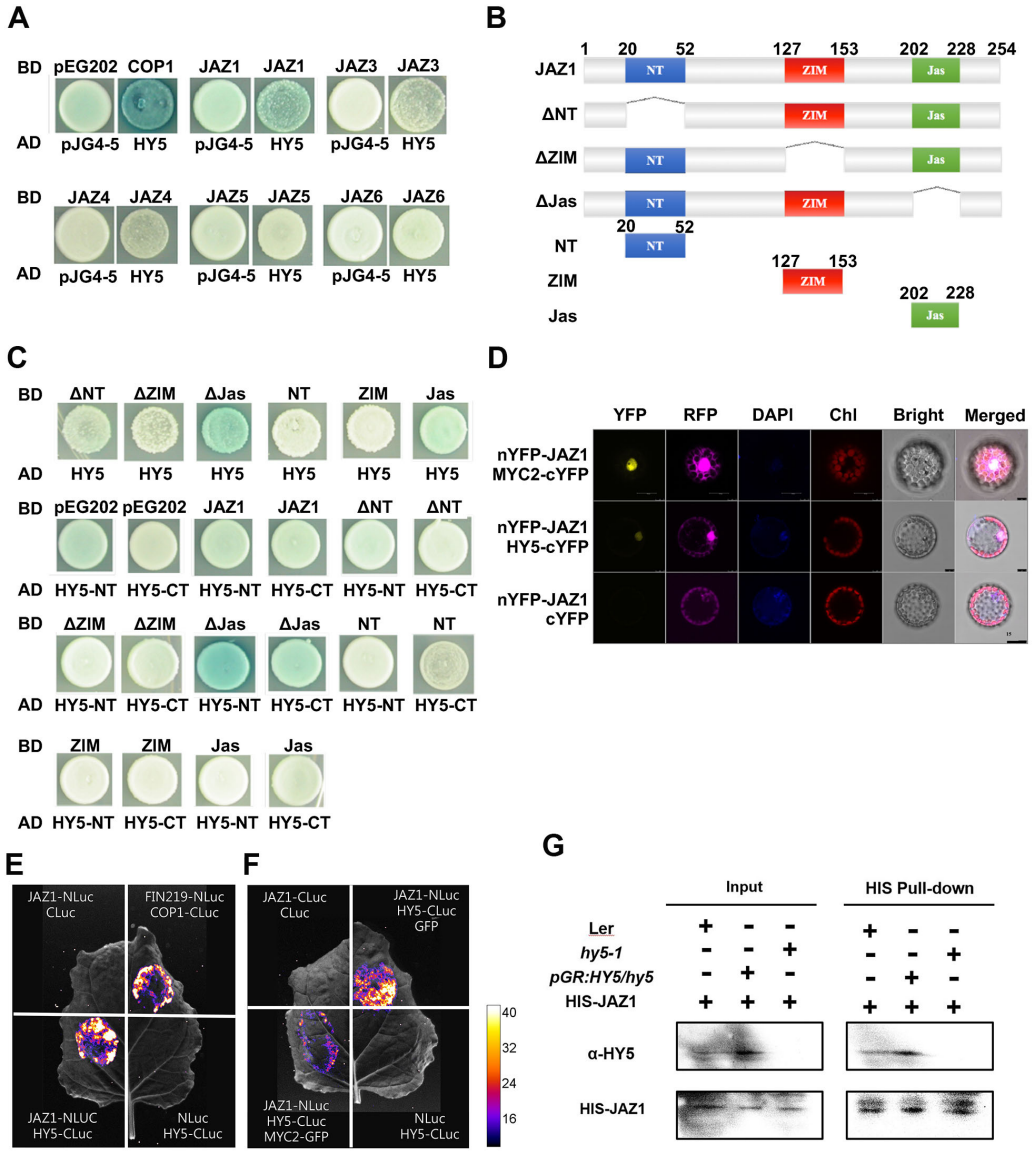
HY5 physically interacts with the JAZ1 protein. **(A)** JAZ1 shows physical interaction with HY5 in yeast two-hybrid assays. *pEG202*-expressed COP1 and *pJG4-5*-expressed HY5 were used as positive controls. Yeasts carrying empty vectors were used as negative controls. Transformed yeasts were screened on SD/Raf/Gal -His-Trp-Leu medium in the presence of X-gal. The COP1 and HY5 combination is used as a positive control. The pEG202 and pJG4–5 combination, along with pEG202 or pJG4–5 alone, serves as a negative control. **(B)** Illustration of the constructs used to examine the critical protein-protein interaction domains on JAZ1. **(C)** The Jas motif may be vital for interaction with HY5. Deleting the Jas motif (ΔJas) leads to increased interaction with full-length HY5 and the HY5 N-terminus. Transformed yeasts were screened on SD/Raf/Gal -His-Trp-Leu medium in the presence of X-gal. **(D)** cYFP-HY5 physically interacts with JAZ1-nYFP in transfected Arabidopsis protoplasts. YFP-JAZ1 and MYC2-cYFP were used as positive controls. nYFP-JAZ1 and cYFP were used as negative controls. Images from the RFP, DAPI, chlorophyll, and bright-field channels were used to show the relative locations of organelles, respectively. Protoplasts were isolated from 4-week-old Col-0 plants. **(E, F)** Luciferase complementation imaging (LCI) assays revealed that HY5 interacts with JAZ1 in *Nicotiana benthamiana* leaves, but MYC2 disrupted this interaction. Full-length JAZ1 and HY5 were fused to the N-terminal (NLuc) and C-terminal (CLuc) fragments of luciferase, respectively. Full-length MYC2 fused to GFP (MYC2-GFP) was used as a competitor. FIN219-NLuc and COP1-CLuc served as positive controls. The experiments were performed with three biological replicates, yielding similar results. **(G)** Semi-co-immunoprecipitation assay confirms HY5 and JAZ1 interaction. The recombinant protein His-JAZ1 purified from *E. coli* expression system was incubated with proteins extracted from FR light-grown seedlings of wild-type *Ler*, *hy5–1* mutant and *HY5* inducible overexpression line (*pGR: HY5/hy5*) and immunoprecipitated with HIS-tag antibodies, the resulting pellets were subjected for gel blot analyses with the use of polyclonal antibodies against HY5 and HIS-tag, respectively.

The EMSA gel shift kit (Affymetrix) was used according to the manufacturer’s instructions. The recombinant proteins (His-HY5, His-JAZ1, CBP-JAZ1-D1A, CBP-JAZ1-D2A, and CBP-JAZ1-D3A) were purified by the corresponding affinity chromatography and validated by Western blotting with specific antibodies. We used His-HY5 at 4 μg and His-JAZ1 at 6 μg in the G-box EMSA tests, and His-HY5 at 4 μg and 1.5 μg of each of the four *JAZ1* variants in EMSA tests of *JAZ1* variants. After gel electrophoresis, EMSA gels were transferred onto Immobilon-Ny blotting membranes (Millipore), and signals were detected using the LAS3000 imaging system (Fujifilm). Three primer pairs targeting three G-box elements in the promoter region of *JAZ1* were synthesized with (hot probe) or without biotin labeling (cold probe). The locations of these G-boxes are shown in **Figure 1A**. **Supplementary Table 3** lists the primer sequences for probe synthesis.

### *JAZ1* promoter analysis with LUC assay

We used the PlantCARE database to predict potential cis-acting regulatory elements in the 1.5-kb putative promoter region of *JAZ* genes upstream of the transcription start sites (Lescot et al., 2002). The putative *JAZ1* promoter was used in a dual-luciferase reporter assay with the transient expression platform of Arabidopsis mesophyll protoplasts. The *proJAZ1:LUC* and *35S:rLUC* were introduced into protoplasts as the reporter and internal control, respectively. As effectors, *JAZ1/pEG100*, *MYC2/pEG100*, and *HY5/pEG100* were co-transformed in all combinations. Each combination used 5 μg of plasmid for each reporter, control, and effector. The empty *pEG100* was added to each reaction to bring the total plasmid to 20 μg. After 16 hrs of cell culture in a cWL growth chamber, the cells were collected by centrifugation at 50 x g for 1 min, and the supernatant was removed. We transferred the cells to an opaque 96-well plate for further fluorescence detection. Then, we used the kit and protocol for the Dual-Luciferase^®^ Reporter Assay System (Promega, E1910) to measure luciferase activity.

### Real-time quantitative PCR

We obtained cDNAs by reverse transcription using the High-Capacity cDNA Reverse Transcription Kit (Applied BiosystemsTM) from total RNA extracted with the LabPrepTM RNA Plus Mini Kit (LPRS100) and quantified by the NanoDrop Spectrophotometer (Thermo Scientific, DN-1000). We performed RT-qPCR with the Bio-Rad PCR machines (CFX-384 and CFX-Connect) and iQTM SYBR^®^ Green Supermix (Bio-Rad) reagents. Gene-specific primers used to quantify the expression levels of *HY5* (AT5G11260), *JAZ1* (AT1G19180), *FIN219* (AT2G46370), and a reference gene, *UBQ10* (AT4G05320), are listed in **Supplementary Table 3**. Each 20 μL reaction contained 10 μL iQTM SYBR^®^ Green Supermix, 1 μL of each forward and reverse primer (10 μM), 7 μL ddH2O, and 1 μL of cDNA template (2.5 ng/μL). Data were collected and analyzed using the corresponding software for the machine, Bio-Rad CFX Manager V3.0, and the relative expression level of target genes to the reference gene, UBQ10, was calculated as follows: Ratio = (E_target_) ^ΔCt,target(calibrator-test)^/(E_reference_) ^ΔCt,ref(calibrator-test)^.

### Statistical analyses

To compare hypocotyl lengths across different light conditions, we conducted one-way ANOVA analyses followed by Fisher’s LSD to assess statistical significance, using p < 0.05 as the threshold. For gene expression analysis, statistical significance was determined by two-way ANOVA with Tukey HSD multiple-comparison testing (p < 0.05).

## Results

### Jasmonates induce HY5 protein levels in a FIN219- and COI1-dependent manner

**Figure 2:**
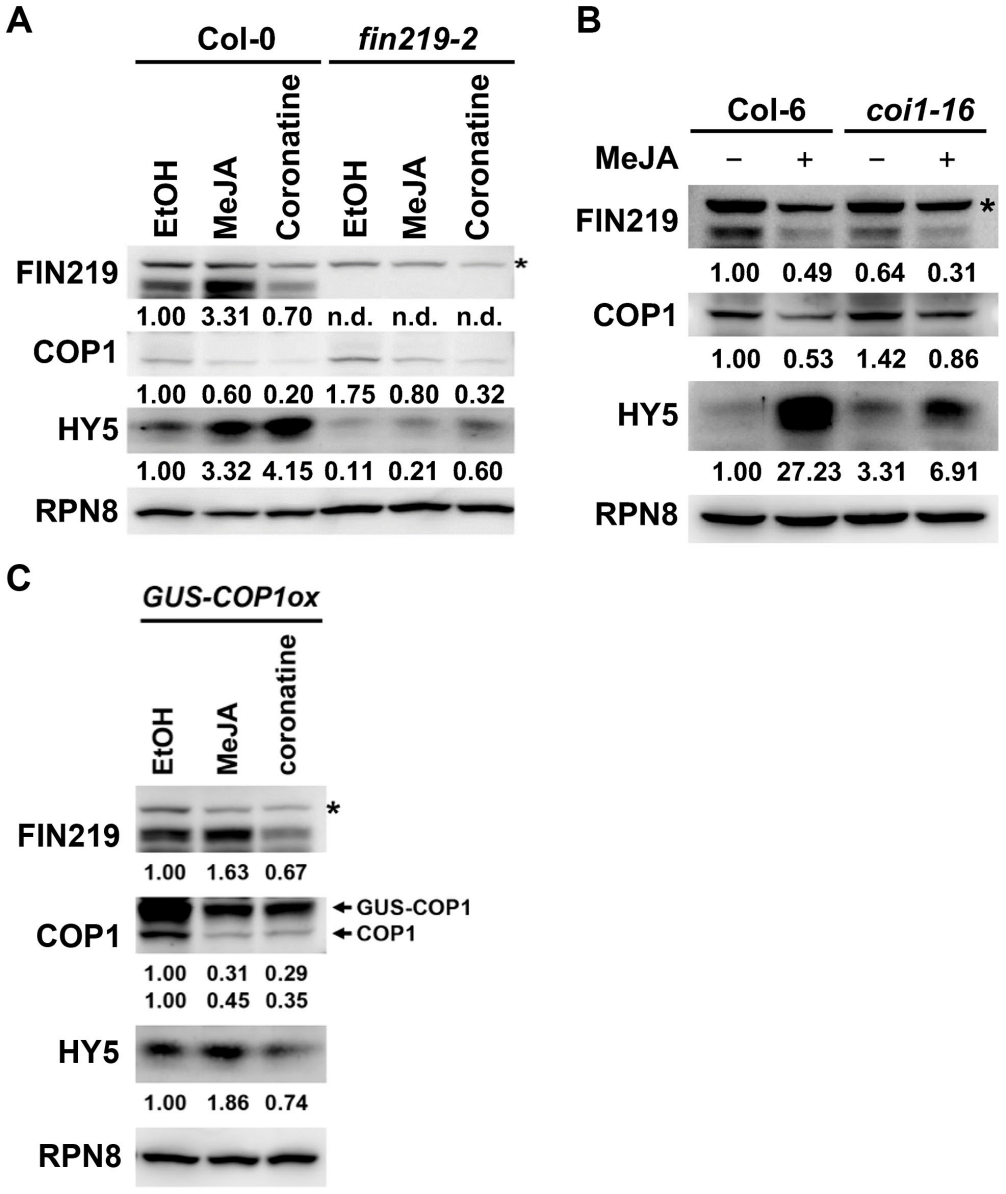
Jasmonates strongly induce HY5 protein levels in a FIN219- and COI1-dependent manner. **(A)** Application of MeJA or coronatine significantly increased HY5 protein levels in a FIN219-dependent manner. Sterilized seeds were sown on GM plates containing 0.1% EtOH, 50 μM MeJA, or 1 μM coronatine. Seedlings were grown under cFR for 3 days and used for total protein extraction and Western blotting. Specific antibodies against FIN219, COP1, HY5, and RPN8 were used for Western blotting. **(B)** MeJA-induced HY5 accumulation requires functional COI1. Protein gel blot analyses of FIN219, COP1, and HY5 levels in Col-6 and coi1–16 mutant under 50 μM MeJA treatment. Seedlings were grown on GM plates containing 0.1% EtOH **(-)** or 50 μM MeJA (+) under cFR for 3 days. The numbers below the blots indicate relative protein levels compared to Col-6 with ethanol (EtOH) treatment, arbitrarily set to 1. **(C)** HY5 induction in Col-0 by MeJA does not act through COP1. The growth conditions were the same as in **(A, B).** The numbers below the COP1 protein blot represent the levels of ectopic transgenic (upper) and endogenous COP1 (bottom), respectively. The asterisk indicates non-specific bands recognized by anti-FIN219 polyclonal antibodies. The numbers below the blots indicate relative protein levels compared to Col-0 **(A)** or *GUS-COP1ox* **(C)** with ethanol (EtOH) treatment, arbitrarily set to 1. RPN8: an internal loading control. There are two biological replicates for protein gel blots, and the results are similar. The blot shown above is one of them. Each biological replicate uses at least 30 seedlings.

The jasmonate-conjugating enzyme FIN219/JAR1 participates in Arabidopsis photomorphogenesis under far-red (FR) light (Staswick and Tiryaki, 2004; Hsieh et al., 2000). Recent studies revealed that FIN219 and the FR photoreceptor phyA antagonize each other through direct interaction under FR light (Jiang et al., 2023). This discovery prompts us to further examine the involvement of JAs in regulating the expression of genes essential for de-etiolation in Arabidopsis seedlings. To test this, we applied methyl jasmonate (MeJA) and the JA-isoleucine (JA-Ile) analog coronatine to Arabidopsis seedlings grown under FR light. Application of MeJA significantly induced FIN219 protein levels in Col-0 under FR light, whereas coronatine did not (**Figure 2A**). Interestingly, MeJA failed to induce FIN219 levels in Col-6 (**Figure 2B**), suggesting ecotype-dependent differential regulation of FIN219 by MeJA. Given that FIN219 negatively regulates COP1 levels through direct interaction under FR light (Wang et al., 2011), we examined whether JAs affect the protein levels of COP1 and HY5 by protein gel blot analyses. MeJA and coronatine treatments reduced COP1 levels in Col-0 and the *fin219–2* mutant (**Figure 2A**). Similar responses were observed in Col-6 and *coi1–16* in response to MeJA treatment (**Figure 2B**). In addition, *fin219–2* and *coi1–16* accumulated higher COP1 levels than their backgrounds, Col-0 and Col-6, respectively, under control EtOH treatment, indicating that both FIN219 and COI1 may negatively regulate COP1 protein abundance (**Figures 2A, B**).

Strikingly, MeJA and coronatine treatments substantially increased HY5 levels in Col-0 by 3.32- and 4.15-fold, respectively, compared with the control under FR light. In contrast, the *fin219–2* mutant largely abolished MeJA- and coronatine-induced HY5 (**Figure 2A**), suggesting that FIN219 is required for HY5 induction by MeJA and coronatine under FR light. Similarly, the JA receptor COI1 was responsible for MeJA-mediated HY5 induction, as MeJA application led to substantial increases in HY5 levels in Col-6. In contrast, induction was significantly suppressed in the *coi1–16* mutant (**Figure 2B**). These results indicate that jasmonate- and coronatine-mediated HY5 induction requires FIN219 and COI1 under FR light.

Since COP1 negatively regulates HY5 protein levels during photomorphogenesis, and FIN219, an extragenic suppressor of COP1, represses COP1 levels through direct interaction under FR light, we speculate that COP1, which is responsible for HY5 induction by MeJA or coronatine in Col-0, would suppress HY5 protein levels when ectopically expressed. Thus, *COP1* overexpression in the Col-0 background (*GUS-COP1ox*) was generated and examined for these three protein levels under FR light in the presence of MeJA or coronatine. The results revealed that FIN219 levels in *GUS-COP1ox* increased with MeJA and decreased with coronatine, compared with the control (**Figure 2C**), consistent with those in Col-0. Moreover, combined GUS-COP1 and endogenous COP1 levels were significantly increased but showed a decreasing trend in response to MeJA and coronatine treatments, similar to those observed in Col-0 (**Figure 2C**). However, HY5 levels in *GUS-COP1ox* still increased in response to MeJA and were slightly reduced by coronatine (**Figure 2C**). Taken together, HY5 induction by MeJA and coronatine in Col-0 is not mediated by COP1 and is strongly affected by FIN219 and COI1.

### The *hy5* mutant shows hypersensitivity to MeJA-mediated inhibition of hypocotyl elongation, dependent on FIN219 under all light conditions

**Figure 3:**
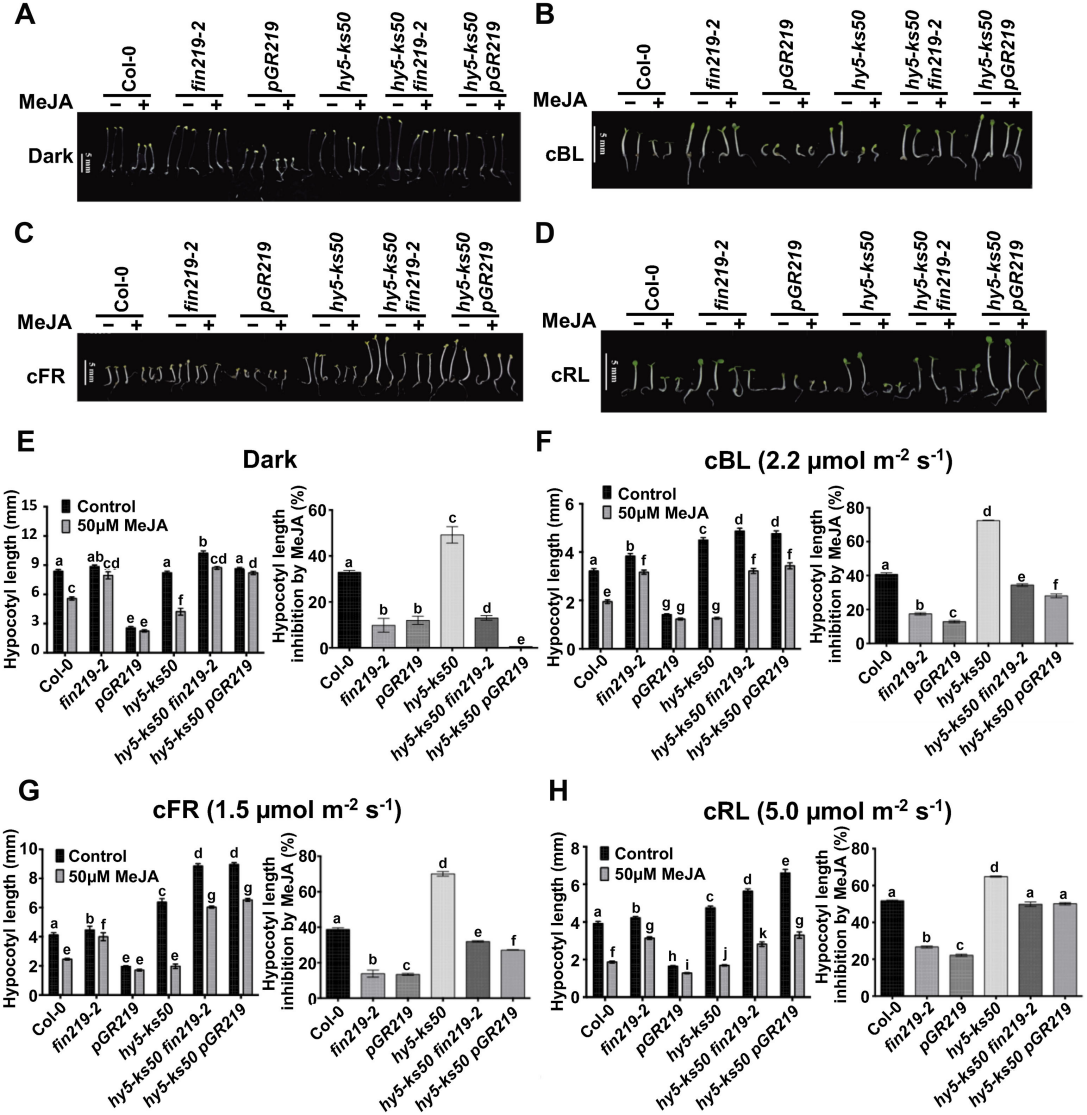
The *hy5* mutant shows hypersensitivity to MeJA-mediated inhibition of hypocotyl elongation, with dependence on FIN219 under all light conditions. **(A-D)** Phenotypes of 10-d-old seedlings of Col-0, *fin219–2* mutant, glucocorticoid-inducible *FIN219* overexpression line (*pGR219*), *hy5* mutant (*hy5-ks50*), *hy5-ks50 fin219-2*, and *hy5-ks50 pGR219* under dark, continuous blue light (cBL), continuous far-red light (cFR), and continuous red light (cRL), respectively. Scale bars indicate 10 mm. **(E-H)** Hypocotyl length measurements indicate that the *hy5* mutant is hypersensitive to MeJA treatment in a FIN219-dependent manner. Error bars indicate SEM from n > 15. Lowercase letters in the figure represent significant differences by one-way ANOVA followed by Fisher’s LSD at p < 0.05.

Given that JAs induce HY5 protein levels under FR light in a FIN219-dependent manner (**Figure 2A**), we examined how HY5, a key regulator of photomorphogenesis, contributes to JA-mediated growth responses, focusing on inhibition of hypocotyl elongation. We generated *hy5-ks50 fin219–2* and *hy5-ks50 pGR219* (*FIN219* inducible overexpression) lines and assessed their hypocotyl responses under constant blue, red, and FR light (cBL, cRL, cFR) and in darkness, with or without MeJA treatment. In the absence of MeJA, *hy5-ks50 fin219–2* displayed a more elongated hypocotyl than either of its parental single mutants, *hy5-ks50* and *fin219-2* (**Figures 3A–H**), suggesting that FIN219 and HY5 synergistically regulate the photomorphogenic response of seedlings upon exposure to light. Furthermore, *hy5-ks50 pGR219* displayed a more elongated hypocotyl than *hy5-ks50* and *pGR219* in darkness (**Figures 3A, E**) and under various light conditions (**Figures 3B–D, F-H**), suggesting that FIN219’s function in photomorphogenesis requires functional HY5 to regulate inhibition of hypocotyl elongation.

Under MeJA treatment, *hy5-ks50* exhibited greater sensitivity to hypocotyl inhibition than Col-0 in darkness (**Figures 3A, E**) and across light conditions (**Figures 3B–D, F–H**). The *hy5-ks50 fin219–2* double mutant responded similarly to *fin219–2* in darkness (**Figures 3A, E**) but showed significantly enhanced MeJA sensitivity under light compared with *fin219-2* (**Figures 3B–D, F–H**). This suggests that the combined loss of HY5 and FIN219 amplifies JA responsiveness beyond that of the loss of FIN219 alone.

Surprisingly, *hy5-ks50 pGR219* was less sensitive to MeJA treatment than its parental lines, *hy5-ks50* and *pGR219*, in darkness (**Figures 3A, E**) and showed intermediate or reduced sensitivity under light (**Figures 3B, C, F–G**), with a response comparable to Col-0 under cRL (**Figures 3D, H**). These findings indicate that the FIN219-mediated JA response requires functional HY5. Together, these results suggest that FIN219 and HY5 mutually influence JA-regulated inhibition of hypocotyl elongation in both light and dark.

### HY5 positively regulates MeJA-mediated anthocyanin accumulation by modulating key transcriptional regulators

HY5 is a central transcription factor in light signaling and regulates anthocyanin biosynthesis (Shin et al., 2007; Qiu et al., 2019). Because jasmonates, including MeJA and coronatine, also promote anthocyanin accumulation under light, we investigated whether HY5 contributes to JA-mediated regulation of this pathway. We first examined anthocyanin accumulation in Col-0 and *fin219–2* seedlings treated with MeJA or coronatine under FR light. Both compounds significantly increased anthocyanin levels in Col-0, but this induction was markedly reduced in *fin219-2* (**Supplementary Figure 1**), indicating that FIN219 is required for JA-induced anthocyanin accumulation.

**Figure 4:**
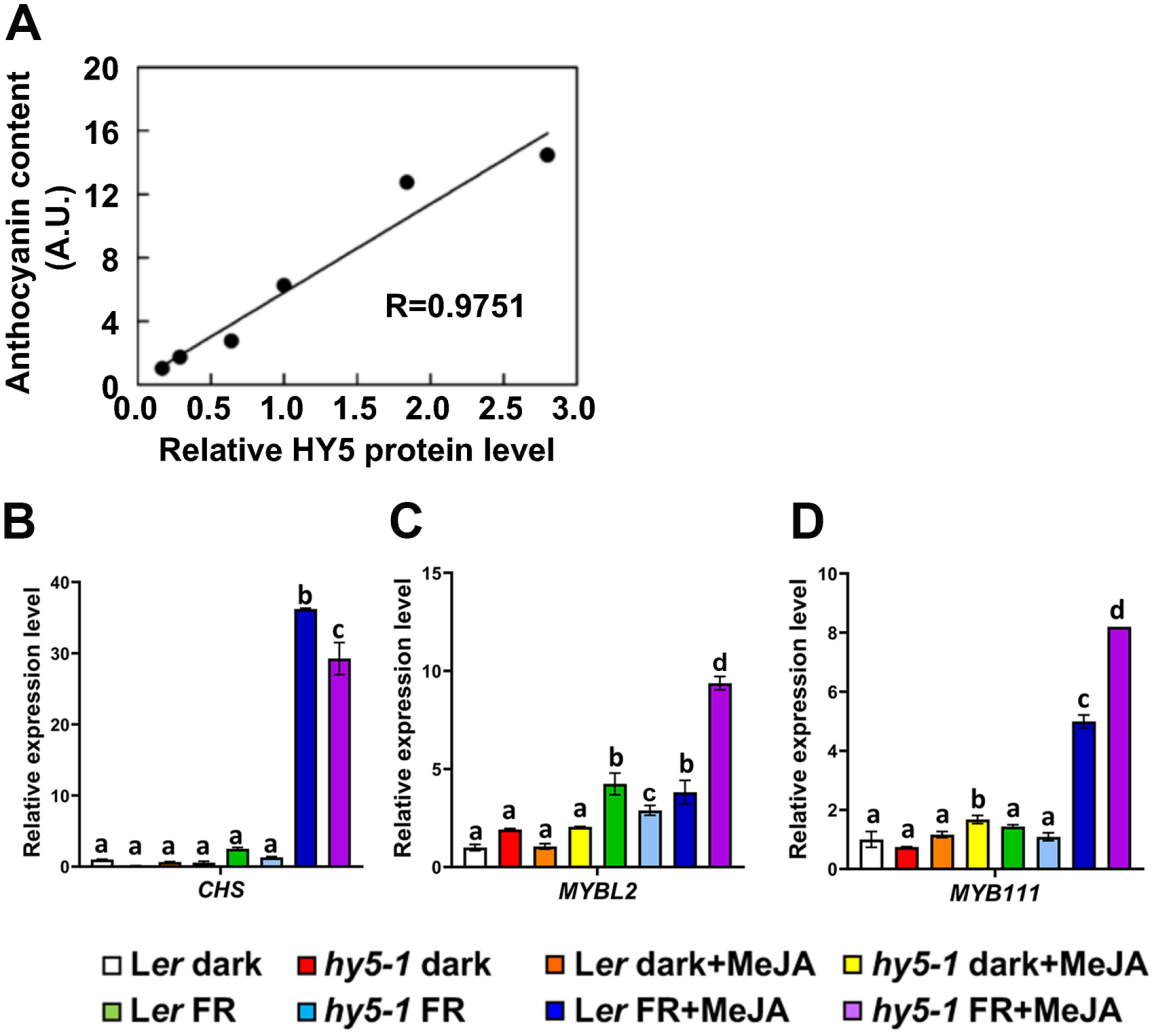
HY5 positively regulates MeJA-mediated anthocyanin accumulation by differentially modulating the expression of key regulators. **(A)** HY5 protein levels are highly correlated with cFR and MeJA-mediated anthocyanin accumulation in seedlings. The correlation analysis was based on anthocyanin content in **Supplementary Figure S1** and relative HY5 protein levels in **Figures 2A, respectively**. **(B-D)** RT-qPCR analysis of essential anthocyanin biosynthetic genes in Ler and hy5–1 mutant under dark, cFR, and MeJA treatments. Relative expression levels of the genes are shown as 2-ddCt relative to UBIQUITIN 10 (UBQ10). The lowercase letters in the figure indicate significant differences by one-way ANOVA followed by Fisher’s LSD at p < 0.05.

We next correlated HY5 protein levels with anthocyanin content under FR light and found a strong positive relationship (**Figure 4A**). Previous studies have indicated that several key transcription factors, including MYBL2 as a negative regulator and MYB111 as a positive regulator, participate in the regulation of anthocyanin biosynthesis (Wang et al., 2016; Hichri et al., 2011). To explore regulatory mechanisms, we quantified the expression of *CHS*, *MYBL2*, and *MYB111* using RT-qPCR in *hy5–1* and wild-type *Landsberg erecta* (*Ler*). Results revealed that *CHS* expression was substantially induced by MeJA and reduced in the *hy5–1* mutant under FR light (**Figure 4B**); *MYBL2* expression was significantly induced in *Ler* and slightly decreased in *hy5–1* under FR light, but showed no induction in *Ler* with MeJA treatment under the same conditions. The *hy5–1* mutant with MeJA showed substantially increased *MYBL2* expression compared to *Ler* with MeJA under FR light (**Figure 4C**). In contrast, *MYB111* expression in *Ler* was significantly induced by MeJA compared to *Ler* without MeJA under FR light, but this induction was even more pronounced in the *hy5–1* mutant with MeJA (**Figure 4D**), suggesting that HY5 negatively regulates *MYB111* expression in the presence of MeJA. Taken together, HY5 controls anthocyanin accumulation by regulating specific transcription factors, and the HY5-mediated regulation of these transcription factors might be altered by MeJA.

### Microarray assays indicate that HY5 participates in regulating the expression of genes involved in FR light and JA signaling

**Figure 5:**
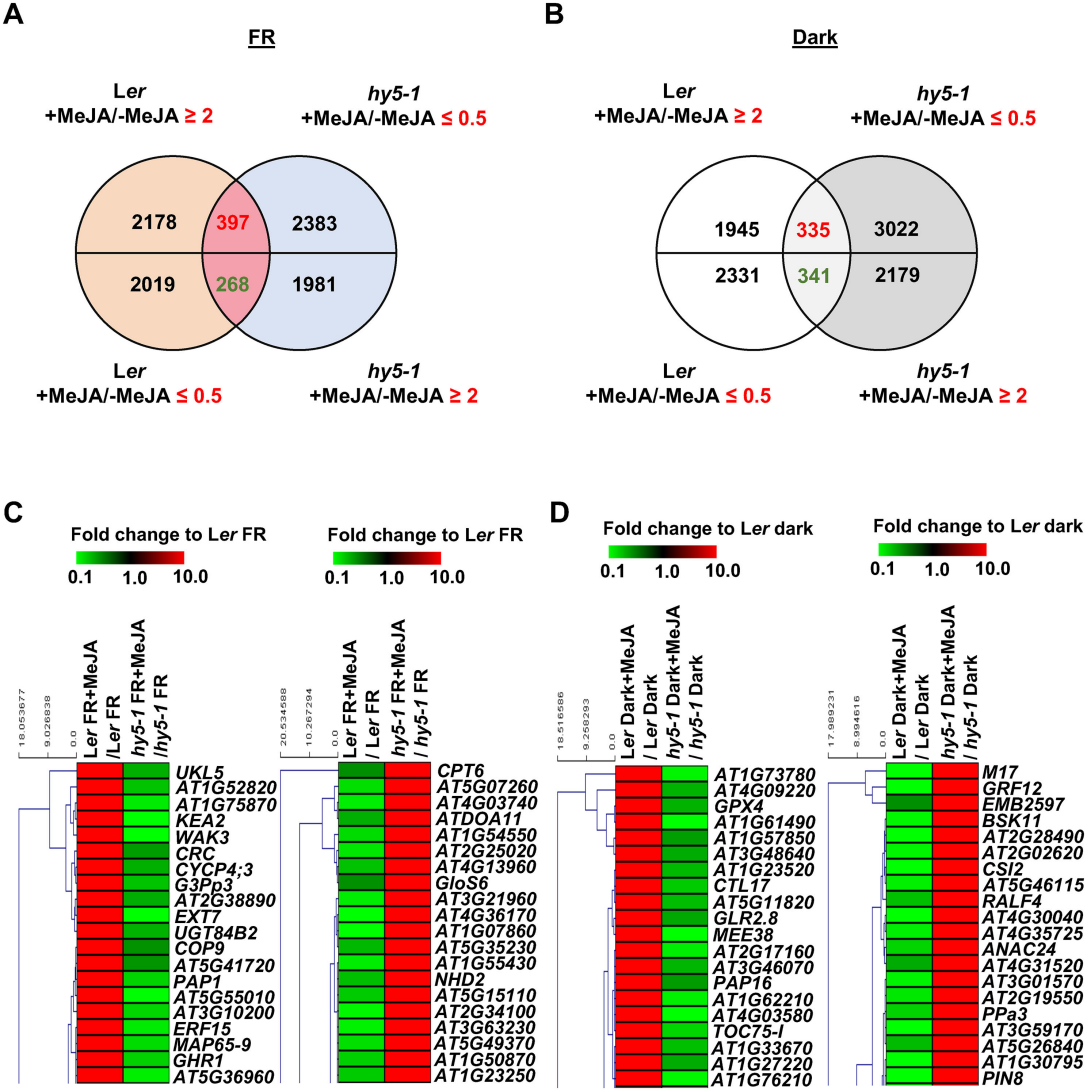
Microarray assays indicate that HY5 regulates cross-talk between FR light and JA signaling during seedling photomorphogenic growth. **(A, B)** MeJA differentially expressed genes were defined as having 2- or 0.5-fold changes in Ler+MeJA/-MeJA control. HY5-dependent genes were defined as having 0.5- or 2-fold changes in *hy5-1*+MeJA/-MeJA control under FR light **(A)** and dark **(B)** conditions, respectively. **(C, D)** Top 20 genes from hierarchical clustering showing *HY5*-dependent MeJA responsiveness under FR **(C)** and dark **(D)** conditions, respectively. In panel **(C)**, the left image shows genes with MeJA-mediated increases in wild-type *Ler* and decreases in the *hy5–1* mutant under FR light. The right image shows genes with MeJA-mediated decreases in wild-type Ler and increases in the *hy5–1* mutant under FR light. In panel **(D)**, the left image shows genes with MeJA-mediated increases in wild-type Ler and decreases in the *hy5–1* mutant under darkness. The right image shows genes with MeJA-mediated decreases in wild-type *Ler* and increases in the *hy5–1* mutant under darkness. Hierarchical clustering was based on Euclidean distances among gene fold changes.

HY5 is a crucial positive regulator of photomorphogenesis, controlling the expression of thousands of light-responsive genes (Lee et al., 2007). Given that MeJA application substantially increases HY5 protein levels under FR light (**Figure 2**) and that the *hy5* mutant is hypersensitive to MeJA treatment under all light conditions, including darkness (**Figure 3**), it is likely that HY5 plays a negative role in regulating JA-mediated responses. Thus, we performed microarray analyses using wild-type *Ler* and the *hy5–1* mutant under FR light with or without MeJA treatment. A total of 2575 genes were up-regulated in *Ler* by at least 2-fold with MeJA versus without MeJA under FR light, while 2287 genes were down-regulated under the same condition (**Figure 5A**). To investigate the importance of HY5 in MeJA-mediated signaling, we further categorized differentially expressed genes into HY5-dependent MeJA-induced and MeJA-suppressed genes. The results indicated that, under FR light, the *hy5–1* mutant with MeJA treatment down-regulated 2780 genes compared with the untreated control. Among them, 397 genes were induced in *Ler* by MeJA in a *HY5*-dependent manner (**Figure 5A**), suggesting that HY5 positively regulates these genes in response to MeJA. In contrast, the *hy5–1* mutant with MeJA, compared with without MeJA treatment, up-regulated 2249 genes under FR light. Among them, 268 genes in *Ler* were down-regulated by MeJA in a HY5-dependent manner (**Figure 5A**), implying that HY5 negatively regulates these 268 genes upon exposure to MeJA. Additionally, jasmonates regulate physiological responses such as adventitious rooting and inhibition of chlorophyll biosynthesis in dark-grown seedlings (Dob et al., 2021; Pan et al., 2021; Sineshchekov et al., 2004; Hsieh and Okamoto, 2014). Further studies indicated that MeJA treatment, compared with no MeJA treatment, led to increased expression of 2280 genes and decreased expression of 2672 genes in dark-grown *Ler* seedlings. In contrast, exogenous MeJA, compared with no MeJA treatment, reduced the expression of 3357 genes in dark-grown *hy5–1* mutant seedlings, in which MeJA induced 335 genes in *Ler* (**Figure 5B**). However, *hy5–1* seedlings with MeJA, compared with those without MeJA, up-regulated 2520 genes, while MeJA decreased 341 genes in *Ler* (**Figure 5B**). Collectively, HY5 regulates a number of genes that modulate certain MeJA-mediated responses under FR light and dark conditions.

Further hierarchical clustering of the top 20 expression candidates affected by MeJA under FR light and darkness, as shown in **Figures 5A, B**, respectively, revealed that exogenous MeJA differentially affects the expression of these genes in a HY5-dependent manner under FR light (**Figure 5C**; **Table 1**) and darkness (**Figure 5D**; **Table 1**). The literature reports some of them, mainly in the control of cell wall organization and ion homeostasis. For example, *WALL ASSOCIATED KINASE 3* (*WAK3*) (He et al., 1999), *COP9*, and *ERF15* (Zhang et al., 2015), which are induced in *Ler* by MeJA and suppressed in the *hy5–1* mutant under FR light, participate in the control of JA signaling or biosynthesis. Additionally, some candidates, such as *CPT6* (*cis-prenyltransferase 6*) (Cunillera et al., 2000), *Glos6* (*GALACTINOL SYNTHASE 6*) (Taji et al., 2002), and *NHD2* (*Sodium/proton antiporter 2*) (Cellier et al., 2004), which are down-regulated by MeJA in *Ler* and markedly up-regulated in the *hy5–1* mutant under FR light, are primarily involved in salt stress responses.

**Table 1:** Enriched gene functions involved in *HY5*-dependent FR and MeJA up- or down-regulated responses.

FR+MeJA up-regulated genes, HY5-dependent		
GO term	Genes	p-value (Fisher's exact test)
Oxidation-reduction process	*AT5G47000*, *CYP76G1*, *ATGA2OX3*, *HSD6*, *CYP704B1*, *CYP96A10*, *CYP76C5*, *NCED5*, *AT1G31670*, *AT1G52800*, *AT1G52820*, *CYP78A10*, *CYP78A9*, *AT1G80320*, *CYP96A9*, *AT1G60730*, *CEPD2*, *FMOGS-OX2*, *AT5G19880*, *AT5G51930*, *AT5G43450*, *AT5G51950*, *hemf2*, *CYP86C4*, *CKX2*, *CYP76C6*, *UCC3*, *ATBBE17*, *CYP86C1*, *AT3G44970*, *CYP703A2*, *GULLO6*	4.90E-04
Positive regulation of transcription, DNA-templated	*DOF4.4*, *MYB83*, *KNATM*, *AT5G18450*, *DREB19*, *ABI5*, *MYB118*, *DREB2C*, *AT2G40350*, *MYB103*	5.40E-04
Sporopollenin biosynthetic process	*CYP704B1*, *CYP703A2*	3.30E-03
Cell differentiation	*FRI*, *MYB83*, *CRC*, *RGF8*, *MYB26*, *PAP1*, *MYB22*, *MYB118*, *MYB53*, *MYB103*	5.30E-03
Regulation of transcription from RNA polymerase II promoter	*MYB83*, *MYB26*, *PAP1*, *MYB22*, *MYB118*, *ALY3*, *MYB53*, *MYB103*	6.60E-03
FR+MeJA down-regulated genes, HY5-dependent		
GO term	Genes	p-value (Fisher's exact test)
Polyprenol biosynthetic process	*CPT9*, *CPT8*, *CPT6*	3.70E-05
Regulation of membrane potential	*CNGC16*, *CNGC11*, *CNGC3*	2.10E-03
Protein glycosylation	*CPT9*, *CPT8*, *CPT6*, *PGSIP7*	3.80E-03
Carbohydrate biosynthetic process	*PGSIP7*, *GAUT2*, *GloS6*	8.30E-03
KEGG pathway	Genes	Fisher Exact
Terpenoid backbone biosynthesis	*CPT9*, *CPT8*, *CPT6*	5.10E-03
Dark+MeJA up-regulated genes, HY5-dependent		
GO term	Genes	p-value (Fisher's exact test)
Signal transduction	*TN13*, *RLP32*, *AT1G33670*, *AT1G63870*, *RLP36*, *AT1G63880*, *AT1G57850*, *AT1G69550*, *RLP25*, *AT5G36930*, *AT2G17050*, *RLP20*, *AT1G47885*, *RLP9*	6.20E-04
SCF-dependent proteasomal ubiquitin-dependent protein catabolic process	*AT1G51320*, *AT3G19880*, *AT3G18330*, *AT3G17280*, *AT3G49510*, *AT3G17490*, *AT4G17200*	1.50E-03
heme transport	*ABCI4*, *ABCI5*	3.40E-03
Ion transmembrane transport	*NIP4;2*, *NIP4;1*, *TIP5;1*	3.80E-03
Regulation of seed dormancy process	*DOG1*, *GSR1*	4.30E-03
Defense response	*BG5*, *AT3G48080*, *TN13*, *RLP32*, *AT1G63870*, *SUMM2*, *RLP36*, *AT1G63880*, *AT1G57850*, *AT1G69550*, *BAP2*, *AT5G36930*, *AT2G17050*, *LRRAC1*, *RKF2*	8.00E-03
Cellular water homeostasis	*NIP4;2*, *NIP4;1*, *TIP5;1*	8.30E-03
Recognition of pollen	*AT1G61430*, *AT1G61480*, *AT1G61490*	9.60E-03
KEGG pathway	Genes	p-value (Fisher's exact test)
Phenylpropanoid biosynthesis	*AT1G24110*, *AT4G25980*, *BGLU28*, *AT5G58390*, *AT5G64110*	7.80E-03
Dark+MeJA down-regulated genes, HY5-dependent		
GO term	Genes	p-value (Fisher's exact test)
Positive regulation of transcription from RNA polymerase II promoter	*AGL45*, *AGL14*, *AGL40*, *AP3*, *AGL71*, *AGL99*, *SEP2*	7.30E-04
MAPK cascade	*AGL14*, *AGL40*, *AP3*, *AGL71*, *SEP2*	9.00E-04
Defense response	*AT5G45220*, *RLP42*, *SNC4*, *AT1G65870*, *CRK23*, *AT4G09430*, *MLO7*, *AT2G32140*, *AT2G15010*, *BG1*, *AT5G47260*, *AT3G44400*, *AT4G16930*, *AT5G35450*, *AT5G28010*, *MLP423*, *AT4G27290*, *LCR80*	1.10E-03
Cell wall modification	*PME48*, *PE11*, *PME19*, *PPME1*, *AT4G15980*	2.00E-03
Negative regulation of histone acetylation	*AT4G04260*, *AHL16*	2.90E-03
Seed oilbody biogenesis	*AT3G01570*, *OLEO2*	2.90E-03
Leaf shaping	*DPA4*, *ZPR3*	3.90E-03
Lipid storage	*AT3G01570*, *AT2G25890*, *OLEO2*	4.10E-03
Pollen sperm cell differentiation	*DAZ2*, *DAF1*, *DAZ3*	5.50E-03
Pectin catabolic process	*PME48*, *PE11*, *PME19*, *PPME1*, *AT4G15980*	6.40E-03
Microtubule-based movement	*AT5G42490*, *AT4G24170*, *TES*, *AT1G18550*	7.40E-03
Cell wall organization or biogenesis	*TBL1*, *AT5G40180*, *TBL21*	8.50E-03
Phenylpropanoid biosynthetic process	*AT1G65870*, *AT1G07740*, *AT1G07730*	8.50E-03
Signal transduction	*AT3G44400*, *AT5G45220*, *AT4G16930*, *PLC7*, *AT3G23270*, *BCHA2*, *RLP42*, *AT4G13820*, *RLP40*, *AT5G54700*, *AT4G09430*, *AT2G32140*	8.70E-03
KEGG pathway	Genes	p-value (Fisher's exact test)
Starch and sucrose metabolism	*SPS4F*, *EMB2729*, *BGLU19*, *TPS3*, *BGLU47*	1.20E-03

*HY5* dependence was defined as genes with >2-fold change in Ler + MeJA/−MeJA but < 0.5-fold in *hy5-1* + MeJA/− MeJA under darkness or FR light conditions.

In contrast, several known factors in wild-type *Ler* were affected by MeJA treatment in darkness and were HY5-dependent (**Figure 5D**; **Table 1**). Among them, GENERAL REGULATORY FACTOR 12 (GRF12) is a 14-3–3 protein that may be involved in guard cell signaling (DeLille et al., 2001). RALF4 was redundant with RALF19 in regulating pollen tube growth (Mecchia et al., 2017). Since JAs regulate pollen germination, pollen tube elongation, and stomatal closure, they may modulate these GRF12- and RALF4/19-regulated phenotypic responses. How JAs regulate GRF12- and RALF4/19-mediated responses through HY5 in the dark remains unclear. Thus, HY5 is vital for MeJA-mediated responses under FR light and in darkness.

### Far-red light enhances jasmonate biosynthesis and signaling pathways in a HY5-dependent fashion

**Figure 6:**
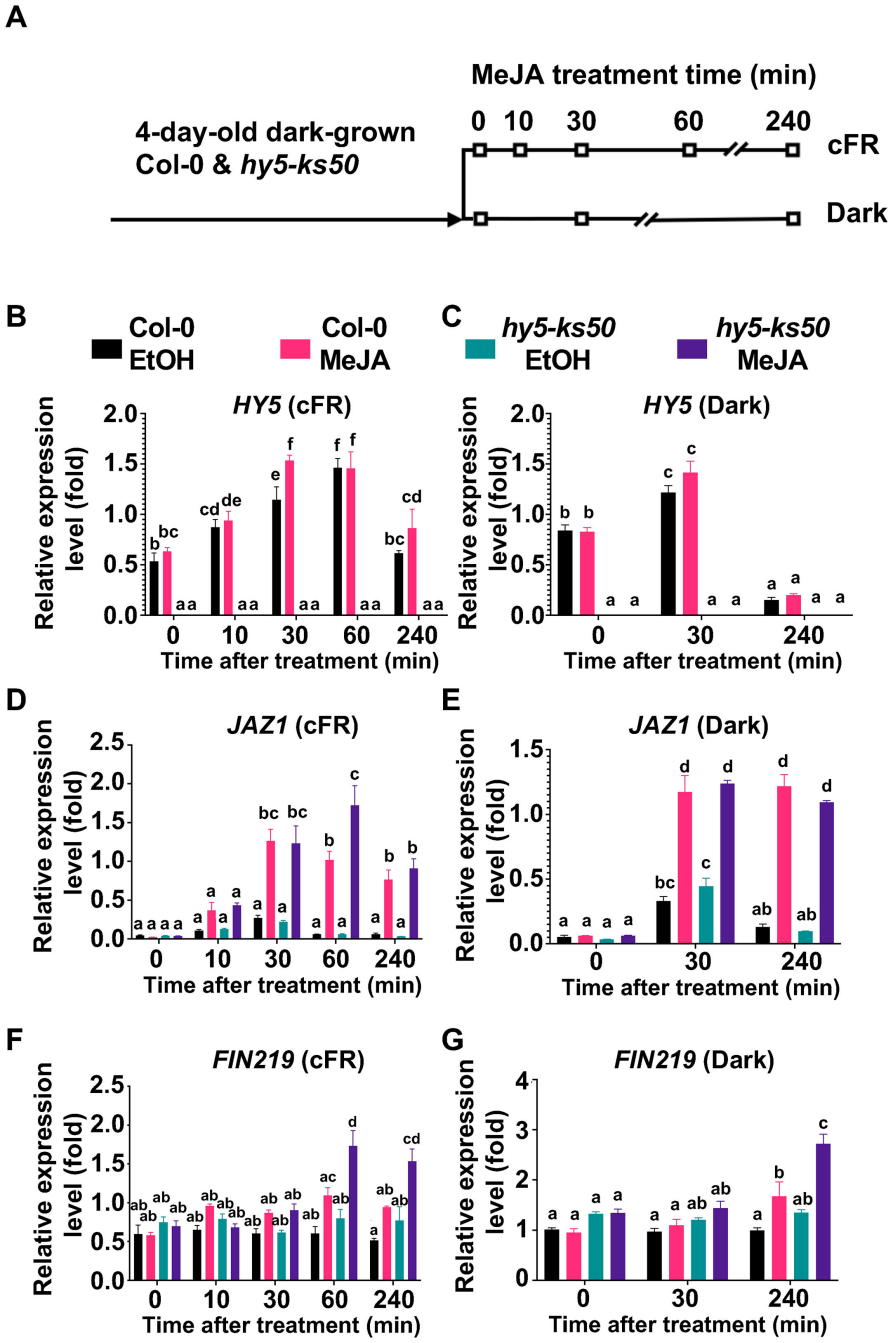
HY5 negatively regulates *JAZ1* gene expression at the transcript level in response to MeJA treatment in a light-dependent manner. **(A)** Illustration of the experimental process for MeJA treatment under dark and cFR. **(B, C)** MeJA triggers the accumulation of *HY5* transcripts under cFR. The MeJA induction of *HY5* precedes that of *JAZ1*. **(D, E)** The MeJA-mediated induction of *JAZ1* is delayed in the *hy5* mutant under cFR. **(F, G)** The levels of *FIN219* in the *hy5* mutant are significantly induced by MeJA under both dark and cFR conditions. Statistical significance was determined by Two-way ANOVA (P < 0.05, n = 3) with Tukey HSD multiple comparisons. The relative expression level (fold) was presented as 2^-ΔCt values relative to *UBQ10*, without further normalization to Col-0 EtOH under dark conditions.

To further examine the effect of HY5 on jasmonate biosynthesis and signaling, we conducted a time-course MeJA treatment on Col-0 and the *hy5-ks50* mutant under continuous FR (cFR) light and dark conditions (**Figure 6A**). cFR irradiation up-regulated *HY5* transcript levels, peaking 1 hr after FR light exposure, whereas MeJA treatment slightly increased *HY5* transcripts at 30 min under FR light (**Figure 6B**). In contrast, MeJA treatment failed to increase *HY5* transcript levels in the dark (**Figure 6C**). These results suggest that jasmonates mainly regulate HY5 in the light, such as FR light, at the protein level rather than at the mRNA level. In addition, MeJA increased *JAZ1* transcript levels in Col-0 under both cFR and dark conditions (**Figures 6D, E**). Interestingly, MeJA-induced *JAZ1* transcript levels were even higher in *hy5-ks50* at 60 min of MeJA treatment than in Col-0 under cFR light conditions, but not in the dark (**Figures 6D, E**), suggesting that HY5 negatively regulates *JAZ1* gene expression at the mRNA level in response to MeJA in a light-dependent manner. In addition, MeJA slightly induced *FIN219* transcript levels in Col-0 at 60 min under cFR (**Figure 6F**), whereas the induction was delayed under dark conditions (**Figure 6G**). Consistent with the observation in Col-0, the *hy5-ks50* mutant also showed significant induction of *FIN219* transcripts at 60 min by MeJA under cFR and at 240 min under dark conditions (**Figures 6F, G**), suggesting that HY5 negatively regulates MeJA-mediated induction of *FIN219* transcript levels under FR light.

**Figure 7:**
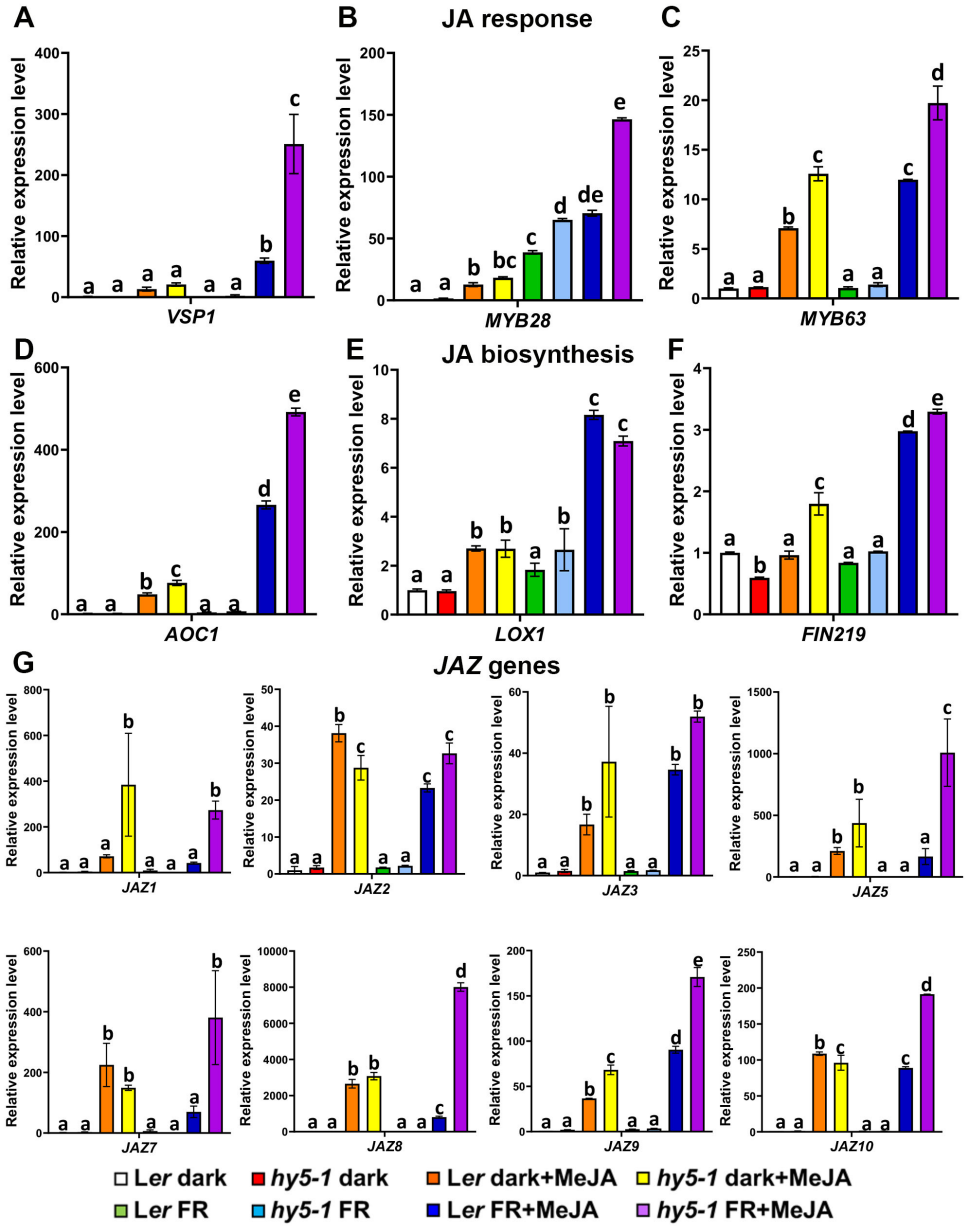
HY5 negatively regulates the expression of JA-responsive and JA-biosynthetic genes under FR light. **(A-C)** Mutation of *HY5* sensitized the expression of JA-responsive genes under dark and cFR light conditions. RT-qPCR validation of JA-responsive genes in Ler and *hy5-1*, with or without MeJA treatment. **(D-F)** MeJA and cFR synergistically up-regulated the expression of JA-biosynthetic genes. **(G)** The *hy5* mutant showed enhanced MeJA induction of *JAZ* genes under cFR. Seedlings were grown on 0.3% GM plates with EtOH (control) or 50 μM MeJA for 4 days under dark or cFR light conditions. Data are shown as mean ± SD. Lowercase letters in the figure represent significant differences by one-way ANOVA followed by Fisher’s LSD at p < 0.05. The relative expression levels of genes, reported as fold changes, were calculated using dCt values relative to UBQ10, then normalized to the dCt value of Ler under dark conditions (set to 1.0) in the absence of MeJA.

To further substantiate the effects of HY5’s functional roles on jasmonate-mediated signaling, we conducted RT-qPCR to examine the expression levels of JA biosynthetic, response, and *JAZ1*-related genes. FR and MeJA treatments synergistically increased transcript levels of *VEGETATIVE STORAGE PROTEIN 1* (*VSP1*), *MYB28*, and *MYB63* (**Figures 7A–C**). Interestingly, the *hy5–1* mutant showed increased expression of these JA-responsive genes in the presence of FR and MeJA compared to Ler with MeJA and *hy5–1* without MeJA (**Figures 7A–C**). Similar expression patterns were observed in the JA biosynthetic genes, such as *ALLENE OXIDE CYCLASE 1* (*AOC1*), *LIPOOXYGENASE 1* (*LOX1*), and *FIN219*, compared to *hy5–1* without MeJA (**Figures 7D–F**), suggesting that HY5 may negatively modulate the expression of JA biosynthetic and responsive genes. Additionally, HY5 negatively regulated the expression of most *JAZ* genes under FR light in the presence of MeJA, except for *JAZ2* and *JAZ3* (**Figure 7G**). At the same time, FR alone failed to induce the expression of all *JAZ* genes examined (**Figure 7G**). However, the *hy5–1* mutant exhibited significantly elevated expression of *JAZ* genes, including *JAZ1*, *JAZ5*, *JAZ7*, *JAZ8*, *JAZ9*, and *JAZ10*, under FR light in the presence of MeJA (**Figure 7G**). Thus, these results reveal that proper induction of light- and JA-responsive genes requires functional HY5, and that HY5 may be required to prevent excessive *JAZ* gene expression and elevated MeJA levels.

### HY5 physically interacts with JAZ proteins

HY5 is a bZIP transcription factor that participates in many signaling pathways, including light and hormone signaling (Ang et al., 1998; Cluis et al., 2004). Primarily, it acts as a critical positive regulator of Arabidopsis photomorphogenic responses. Here, we show its differential roles in regulating JA-mediated inhibition of hypocotyl elongation and JA-induced anthocyanin accumulation (**Figure 3**; **Figure 4**). Interestingly, HY5 also significantly suppresses JA-induced expression of *JAZ* genes in the presence of MeJA under FR light conditions (**Figure 7G**). We wonder whether the adverse effects of HY5 on *JAZ* expression involve a direct protein-level interaction that regulates jasmonate signaling. We performed yeast two-hybrid (Y2H) assays to test this possibility of physical interactions between HY5 and JAZs. Among all the *JAZ*s tested, JAZ1 showed a strong interaction with HY5 (**Figure 1A**). To further investigate the functional domains required for the JAZ1-HY5 interaction, we constructed JAZ proteins with deletions in the NT (ΔNT), ZIM (ΔZIM), and Jas (ΔJas) motifs, respectively (**Figure 1B**). The ΔNT and ΔZIM proteins failed to interact with HY5, suggesting that these two motifs are involved in the proper interaction between JAZ1 and HY5 (**Figure 1C**). Interestingly, the ΔJas enhanced its interaction with HY5, suggesting that the Jas motif may prevent HY5 and JAZ from interacting under certain conditions (**Figure 1C**). Furthermore, ΔJas interacted with the N-terminal region of HY5 (HY5-NT) rather than the C-terminal region of HY5 (HY5-CT) (**Figure 1C**). We also expressed the respective NT, ZIM, and Jas motifs of JAZ1. Still, neither of them could interact with HY5 (**Figure 1C**). To verify the JAZ1-HY5 interaction, we conducted bimolecular fluorescence complementation (BiFC) assays. The nYFP-JAZ1 and HY5-cYFP proteins could interact with each other. In contrast, the JAZ1-MYC2 interaction, used as a positive control, was also detected (**Figure 1D**).

To substantiate the interaction between JAZ1 and HY5, further Luciferase complementation imaging (LCI) assays revealed that *JAZ1-NLuc* and *HY5-CLuc* co-expressed in Nicotiana leaves showed LUC signals under light conditions, and the positive control, FIN219-NLuc and COP1-CLuc, did as well, whereas negative controls such as JAZ1-NLuc plus CLuc and NLuc plus HY5-CLuc did not (**Figure 1E**), suggesting that JAZ1 interacts with HY5 in the light. Previous studies reported that JAZ1 interacted with MYC2 (Withers et al., 2012). We wondered whether MYC2 affects the JAZ1-HY5 interaction. We further examined the effect of MYC2 on the JAZ1-HY5 interaction. The results indicated that the LUC signals from *JAZ1-NLuc* and *HY5-CLuc* co-expression were substantially repressed in the presence of MYC2-GFP (**Figure 1F**), implying that the JAZ1 and HY5 interaction may be suppressed by competitive association between JAZ1 and MYC2. Further, a semi-co-immunoprecipitation assay was used to confirm the JAZ1 and HY5 interaction by using the recombinant protein HIS-JAZ1 purified from an *E. coli* expression system, and protein extracts derived from wild-type *Ler*, *hy5–1* mutant, and *HY5* overexpression line (*pGR: HY5/hy5*) grown in FR light. The results revealed that HY5 from *Ler* and *pGR: HY5/hy5* indeed interacted with HIS-JAZ1 protein, but the *hy5–1* mutant failed to show any detectable interaction signal (**Figure 1G**). In summary, HY5 can physically interact with JAZ1, which may be responsible for integrating FR light and jasmonate signaling.

### HY5 represses *JAZ1* expression by binding to *JAZ1’s* promoter region

**Figure 8:**
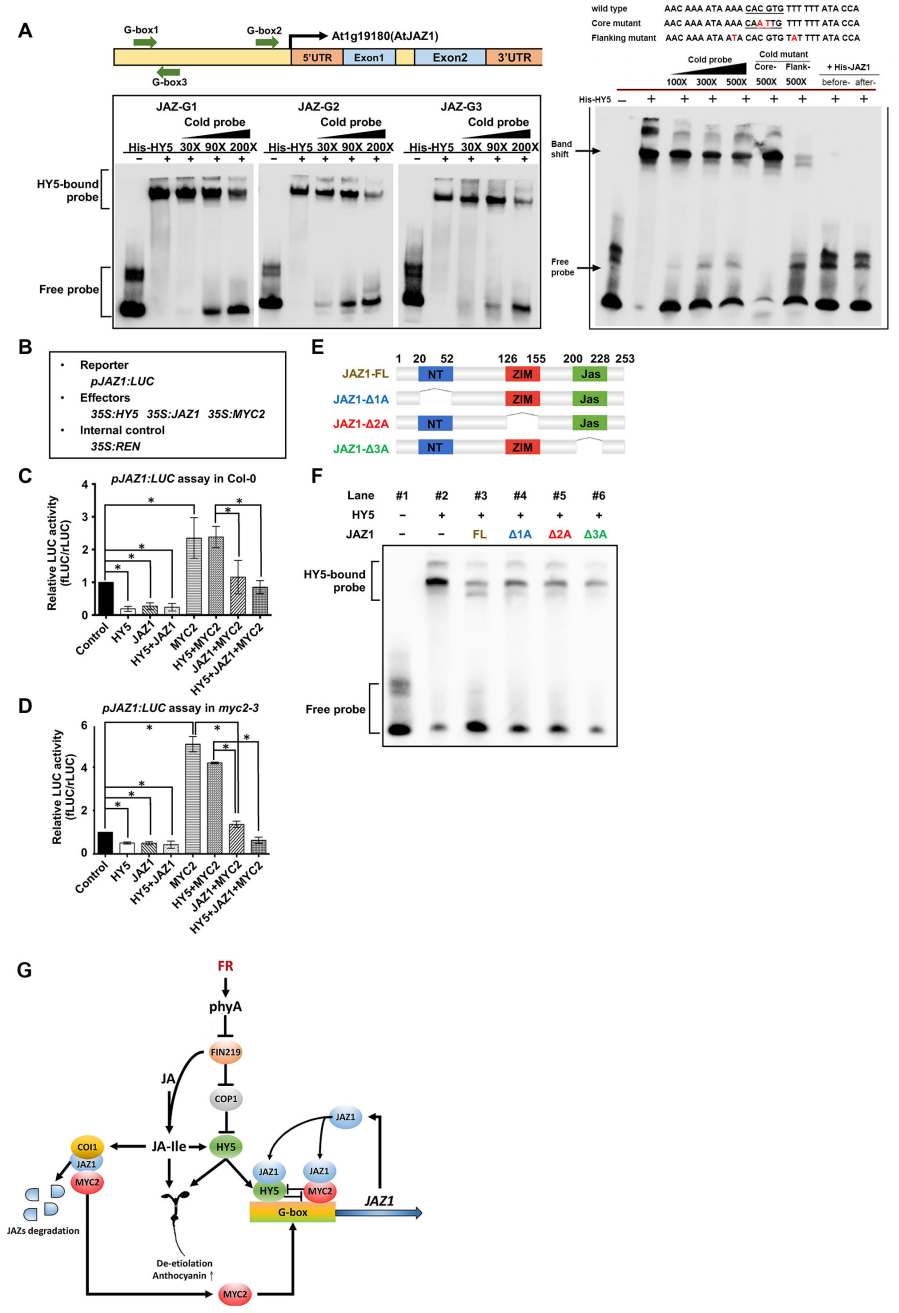
HY5 represses *JAZ1* expression by binding to its promoter region. **(A)** HY5 binds to the G-boxes present in the *JAZ1* promoter. A schematic diagram shows the distribution of the G-boxes in the *JAZ1* promoter. EMSA assays indicate that HY5 binds all three G-boxes in the *JAZ1* promoter. +/- symbols indicate the presence/absence of His-HY5 protein (left panel). Wild-type and mutated forms of G-box2 and recombinant proteins His-JAZ1 were also used in EMSA assays, as shown in the right panel. Four μg of His-HY5 and 10 ng of hot probe were used in each reaction. Different concentrations of cold probes or 4 μg of His-JAZ1 were added for competition assays. **(B)** The experimental layout shows the constructs used in the dual-luciferase transcriptional regulation assays. **(C, D)** HY5 represses the transcription of proJAZ1::LUC in protoplasts isolated from Col-0 **(C)** and *myc2–3* leaves **(D),** while the addition of MYC2 reactivates the transcription of *JAZ1*. Data are shown as mean ± SD from 2 biological replicates. One-way ANOVA with Fisher’s LSD (p < 0.05) was conducted for statistical analyses. **(E)** Illustration of the JAZ1-FL and JAZ proteins with truncations in the N-terminal domain (JAZ1-Δ1A), ZIM domain (JAZ1-Δ2A), and Jas domain (JAZ1-Δ3A), respectively. **(F)** JAZ1 protein interrupts the binding between HY5 and the G-box in the *JAZ1* promoter. EMSA results indicate that adding JAZ1 decreases the signals of HY5-bound G-box probes. **(G)** The proposed model illustrates the functions of HY5 in regulating FR light and JA signaling pathways. HY5 acts as a positive regulator of photomorphogenesis and participates in JA-mediated responses, including suppression of hypocotyl elongation (de-etiolation) and enhancement of anthocyanin accumulation, and is dependent on FIN219/JAR1 and the JA receptor COI1. Moreover, HY5 suppresses the expression of *JAZ1* by directly binding to the G-box cis-elements of the *JAZ1* promoter. It reduces its binding affinity for the target via a feedback loop involving interaction with the JAZ1 protein, leading to JA responses and photomorphogenesis.

HY5 is a key transcription factor that regulates light-responsive gene expression by binding to G-box elements in promoter regions (Ang et al., 1998; Lee et al., 2007; Chattopadhyay et al., 1998). Given the elevated expression of *JAZ* genes in the *hy5–1* mutant (**Figures 6D**, **7G**), we hypothesized that HY5 might transcriptionally regulate *JAZ* gene expression. Cis-element analysis of the putative *JAZ1* promoter region (a 1.5-kb fragment upstream of the transcriptional start site) revealed three G-box motifs (**Figure 8A**; **Supplementary Table 1**). Notably, the putative promoters of other *JAZ* genes, including *JAZ2*, *JAZ3*, *JAZ5*, *JAZ6*, *JAZ7*, *JAZ8*, *JAZ9*, and *JAZ10*, also contained at least one G-box cis-element (**Supplementary Table 1**).

We further examined the possibility of HY5 binding to the G-boxes of the *JAZ1* promoter using electrophoretic mobility shift assays (EMSA). The results indicated that HY5 binds to all three G-box elements of the *JAZ1* promoter, as demonstrated by competitive inhibition with cold probes (**Figure 8A**, left panel). To further confirm HY5’s specific binding to the G-box cis-elements, we included mutant forms of the G-boxes, such as the Core mutant containing mutated nucleotides in the core sequence of G-box2 and the Flanking mutant containing mutated nucleotides in the flanking sequences of G-box2, in competitive binding assays (**Figure 8A**, right panel). The Core mutant competed with the probe for binding to His-HY5, similar to the cold probes. Moreover, the Flanking mutant substantially decreased the association between the probe and His-HY5. Additionally, His-JAZ1 addition abolished His-HY5 binding to the probe, likely through an interaction between His-JAZ1 and His-HY5 (**Figure 8A**, right panel). Overall, HY5 binds specifically to the G-boxes in the *JAZ1* promoter.

To further assess the regulatory effect of HY5 binding on *JAZ1* promoter activity, we performed a dual-luciferase assay in which the *JAZ1* promoter drove luciferase (LUC) expression (**Figure 8B**). As expected, transient *MYC2* expression strongly activated *proJAZ1::LUC*. In contrast, co-expression of either HY5 or JAZ1 significantly suppressed LUC activity. Because JAZ1 did not bind to its own promoter, these results suggest that HY5 acts as a transcriptional repressor of *JAZ1* expression and that *JAZ1* expression may be subject to negative feedback regulation (**Figure 8C**).

To validate this repression mechanism, we repeated the assays in a *myc2–3* null mutant to eliminate background LUC activity from MYC2, a key positive TF for *JAZ1* expression. In *myc2-3*, HY5 significantly repressed *proJAZ1*-driven LUC activity, confirming MYC2-independent repression (**Figure 8D**). Intriguingly, HY5 co-expression suppressed MYC-mediated induction in both Col-0 and *myc2–3* backgrounds (**Figures 8C, D**). Alternatively, the presence of MYC2 may interfere with HY5 repression of *JAZ1* promoter activity through its interaction with HY5 and its binding to the *HY5* promoter (**Figure 8C**; Chakraborty et al., 2019). To further explore whether the JAZ1-HY5 protein interaction affects HY5 binding to G-box elements of *proJAZ1*, we included JAZ1 protein variants with deletions in the NT domain (JAZ1-Δ1A; Δ1A), ZIM domain (JAZ1-Δ2A; Δ2A), and Jas domain (JAZ1-Δ3A; Δ3A) and tested them by EMSA (**Figures 8E, F**). The results suggested that pre-incubation with JAZ1 protein variants reduced HY5’s binding affinity for the G-box elements, while no significant difference was observed among the JAZ1 variants (**Figure 8F**). Overall, HY5 suppresses *JAZ1* expression by binding to its promoter and may reduce MYC2-mediated induction of *JAZ1*.

## Discussion

Crosstalk between light and JA signaling pathways is emerging as critical for regulating JA-mediated responses, including seedling development. In particular, transcription factors such as MYC2 and PIF5 integrate light and JA signaling (Gangappa et al., 2010; Yamashino et al., 2013). Here, we report that JA and coronatine substantially induce HY5 protein levels in FIN219/JAR1- and COI1-dependent manners. Moreover, the *hy5* mutant shows hypersensitivity to MeJA-mediated inhibition of hypocotyl elongation. Genetic studies reveal that FIN219 and HY5 synergistically regulate hypocotyl elongation in response to light treatment (**Figure 3**). Intriguingly, HY5 protein levels strongly correlate with MeJA-enhanced anthocyanin accumulation. In addition to FIN219-dependent inhibition of hypocotyl elongation, HY5, together with suppression of MYC2 function, negatively regulates expression of several *JAZ* genes, such as *JAZ1*, likely by binding to the G-box cis-elements of their promoters, thereby further fine-tuning JA signaling via a feedback mechanism involving HY5 and JAZ1 interaction (**Figure 8G**). Collectively, our findings reveal a new mechanism by which HY5 positively regulates photomorphogenesis by repressing the promoter activity of *JAZ* genes such as *JAZ1* and by modulating the MYC2-mediated induction of *JAZ1* expression, thereby regulating Arabidopsis seedling development.

### Natural variation in FIN219 and in HY5-regulated responses across ecotypes

FIN219, a JA-conjugating enzyme, has been shown to participate in light and hormone signaling pathways (Hsieh et al., 2000; Chen et al., 2015). Here, we further demonstrate that MeJA treatment differentially regulates FIN219 and HY5 protein levels in the Col-0 and Col-6 ecotypes (**Figure 2**), suggesting that these two accessions may have distinct effects on stress responses, including salinity and defense responses. Previous reports indicated that various Arabidopsis accessions differ in the lipid-to-protein ratio of the thylakoid membrane and in pigment distribution, thereby enabling photosynthetic adaptation to diverse environments (Wójtowicz and Gieczewska, 2021). Moreover, natural variation among Arabidopsis accessions contributes to changes in gene expression profiles in response to exogenous salicylic acid (van Leeuwen et al., 2007). The Col-0 ecotype is even sensitive to GA doses, supporting the conclusion that DELLA activity is required for pollen development and seed fertility (Plackett et al., 2014). Here, we report that FIN219 and HY5 protein levels are differentially regulated in Col-0 and Col-6, likely reflecting differences in JA-mediated responses, consistent with the results shown in **Figure 2**.

### Multifaceted roles of HY5 and FIN219 in the regulation of seedling development and hormonal crosstalk

HY5 is a bZIP transcription factor and a pivotal regulator of plant growth and development, light signaling, hormone signaling (Cluis et al., 2004), and stress responses (Kindgren et al., 2012). The *hy5* mutant was initially identified for a root growth defect and as an extragenic suppressor of the *cop1–6* mutant (Oyama et al., 1997; Ang and Deng, 1994). Further studies have established its role as a positive regulator of photomorphogenesis in Arabidopsis and revealed a strong regulatory relationship with COP1, a repressor of photomorphogenesis in darkness (Ang et al., 1998). However, COP1-mediated destabilization of the HY5 protein depends on functional FIN219 (Wang et al., 2011). We further demonstrated that MeJA and coronatine substantially induced HY5 protein levels in FIN219/JAR1- and COI1-dependent manners, but not through COP1 (**Figure 2**). Moreover, the *hy5* mutant is hypersensitive to MeJA-mediated inhibition of hypocotyl elongation across all light conditions, including darkness, suggesting that HY5 may suppress specific genes that modulate the hypocotyl response to MeJA. MeJA up-regulates genes involved in oxidation-reduction processes, and several transcription factors, such as MYB118, MYB83, and ABI5, regulate transcription and sporopollenin biosynthesis in a HY5-dependent manner under FR light conditions (**Table 1**). Interestingly, MYB83 and MYB46 are functionally redundant in regulating secondary wall biosynthesis (Ko et al., 2014). ABI5 has been shown to crosstalk with JA signaling through MED25. MED25 positively regulates JA signaling through its interaction with MYC2. MED25 also enhances ABI5 degradation (Chen et al., 2012). Thus, how HY5 regulates these transcription factors to control seedling development in a JA-dependent manner is intriguing and remains elusive.

It is well known that the *HY5* mutation results in an elongated hypocotyl phenotype under all light conditions (Oyama et al., 1997). Our results indicated that the *fin219–2* mutant exhibited elongated hypocotyls under all tested light conditions, particularly in the presence of MeJA. Interestingly, the *hy5ks50 fin219–2* double mutant had a substantially longer hypocotyl than either *hy5ks50* or *fin219–2* single mutants under cFR (**Figure 3C**), suggesting that FIN219 and HY5 synergistically regulate photomorphogenic development of seedlings under FR light and JA conditions. Moreover, *hy5ks50 pGR219* showed a hypocotyl phenotype similar to that of *hy5ks50 fin219–2* under light conditions (**Figure 3**), implying that FIN219 function requires HY5 to regulate photomorphogenesis in response to light. The current study also reveals that MeJA-induced HY5 depends on FIN219 (**Figure 2**). Thus, FIN219 and HY5 may mutually regulate each other in the light and JA signaling pathways. Primarily, HY5 acts as a positive master regulator in photomorphogenesis by suppressing JAZ1 function in JA signaling and inhibiting hypocotyl elongation in light. We also observed that HY5 controlled stress-responsive TFs and prenyltransferases under FR light (**Figure 5****;** **Table 1**). The synergy between FIN219 and HY5 is likely critical for light-mediated defense responses. Future studies on the molecular interactions between FIN219 and HY5 might reveal a novel regulatory mechanism that tethers light and JA signaling pathways.

### MeJA-mediated changes in HY5 levels regulate anthocyanin accumulation

HY5 has been shown to play a vital role in regulating light-induced anthocyanin accumulation (Shin et al., 2007; Qiu et al., 2019). MeJA also significantly enhances anthocyanin content, especially under stress conditions (Kuang et al., 2025). Here, we further show that MeJA and coronatine substantially increase HY5 protein levels in a FIN219- and COI1-dependent manner (**Figure 2**). Moreover, HY5 positively regulates *MYBL2* expression under FR light; however, MeJA treatment results in a 2-fold increase in *MYBL2* expression in the *hy5–1* mutant (**Figure 4**), suggesting that HY5 negatively regulates *MYBL2* expression in the presence of MeJA. Previous studies indicated that a MYB-like Domain transcription factor (MYBD) induced by light and cytokinin could enhance anthocyanin biosynthesis by repressing MYBL2 (Nguyen et al., 2015). MYBD also positively regulates the expression of genes such as *DFR*, *LDOX*, and *UF3GT*, which are downstream of anthocyanin biosynthesis (Nguyen et al., 2015). Intriguingly, HY5 directly binds to the *MYBD* promoter to positively control its expression (Nguyen et al., 2015). Moreover, the *hy5–1* and *phyB* mutants show significantly lower levels of *MYBD* transcripts than the wild type. Our recent results have revealed that the addition of MeJA substantially enhances phyB-associated photobody formation under shade conditions, and that the *fin219–2* mutant alters the patterns of phyB photobodies, with a reduction in phyB function under ambient light conditions (Peng et al., 2023). In addition, MeJA treatment significantly increases HY5 protein levels (**Figure 2**), and FIN219-mediated JA responses, such as inhibition of hypocotyl elongation, require functional HY5 (**Figure 3**). It is possible that the substantial increase in *MYBL2* transcript levels in the *hy5–1* mutant with MeJA treatment under FR light may be through the combined effects of decreased MYBD and phyB functions. Thus, the molecular mechanisms underlying MeJA-mediated changes in HY5 effects on MYBD and MYBL2 functions in regulating anthocyanin biosynthesis warrant further investigation.

### HY5 and MYC2 compete for binding to the *JAZ1* promoter in JA signaling

This study shows that HY 5 and MYC2 compete for binding to the G-BOX cis-element in the *JAZ1* promoter. HY5 negatively regulates *JAZ1* expression, whereas MYC2 acts as a positive regulator of *JAZ1* expression (**Figure 8**). Competitive binding is a critical mechanism for regulating gene expression and serves as a module to appropriately restrict plant growth and development. The HY5-PIF1 regulatory module is essential for controlling photosynthesis genes in response to changes in light and temperature (Toledo-Ortiz et al., 2014). The competitive regulation of HY5 and MYC2 on JAZ1 may act similarly to fine-tune the expression of downstream light- and JA-responsive genes under normal growth conditions, thereby promoting confirmatory photomorphogenesis in seedlings. In addition, the interaction between HY5 and MYC2 may help relieve suppression of *JAZ1* expression (Chakraborty et al., 2019). In contrast, rapid changes in gene expression may occur in response to environmental stimuli. Furthermore, the positive role of FIN219 in promoting HY5 protein accumulation in response to JA provides an additional layer of regulation of photomorphogenic growth (**Figures 2**, **8G**). *HY5* mutation increased sensitivity to MeJA, particularly the MeJA-mediated inhibition of hypocotyl growth, resulting in an even shorter hypocotyl phenotype than Col-0 under all light conditions (**Figure 3**). However, Yi et al. (2020) reported that the *hy5-205* mutant exhibited an extended hypocotyl phenotype compared to the wild type in the presence of MeJA under all light conditions examined. This discrepancy may be attributed to differences in sucrose concentrations, as their study used 2%, whereas ours used 0.03% (Wang et al., 2011). The interaction of light and sucrose concentration substantially affects hypocotyl elongation. In addition, our results revealed that inducing HY5 in the presence of JA or JA-Ile may serve as a feedback mechanism to prevent exaggerated JA responses that severely repress plant growth.

### The HY5–JAZ–MYC2 network might be critical to plant defense and growth trade-offs

A recent study also suggests that attenuating HY5 DNA-binding activity reduces plant immunity and resistance to the oomycete pathogen *Hyaloperonospora arabidopsidis* (Chen et al., 2021). Moreover, MYC2, MYC3, and MYC4 target the *HY5* promoter to activate its expression, thereby promoting phytochrome-mediated photomorphogenesis (Ortigosa et al., 2020; Yi et al., 2020). Accordingly, interactions between JAZs and HY5, or between JAZs and MYC2, may help maintain balanced growth. In particular, MYC2 levels modulate the JAZ1-HY5 interaction in light (**Figures 1E, F**), which may underlie trade-offs between plant growth and defense. At the same time, rapid degradation of JAZs under JA conditions enables a swift response to biotic stresses. Alternatively, HY5 acts as a critical regulator of photomorphogenesis, working with FIN219 to suppress JAZ abundance at the transcriptional and translational levels, thereby enhancing inhibition of hypocotyl elongation in response to light (**Figure 8G**). Future studies on HY5 functions in defense responses will elucidate the complex crosstalk between light and JA signaling pathways, which is essential for crop improvement in response to changing climates and increasing pest infestation.

## Data Availability

The original contributions presented in the study are included in the article/**Supplementary Material**. Further inquiries can be directed to the corresponding author.
