# Supplementary material for: Arabidopsis HY5 enhances jasmonate responses by directly suppressing the expression of the JAZ1 gene in a FIN219/JAR1-dependent manner
Source: Front Plant Sci. 2026 Aug 3;17:1891433. doi: 10.3389/fpls.2026.1891433 (PMC13478363; doi:10.3389/fpls.2026.1891433)
Supplement: Supplementary file 1 [file DataSheet1.pdf]

Supplemental Data Appendix

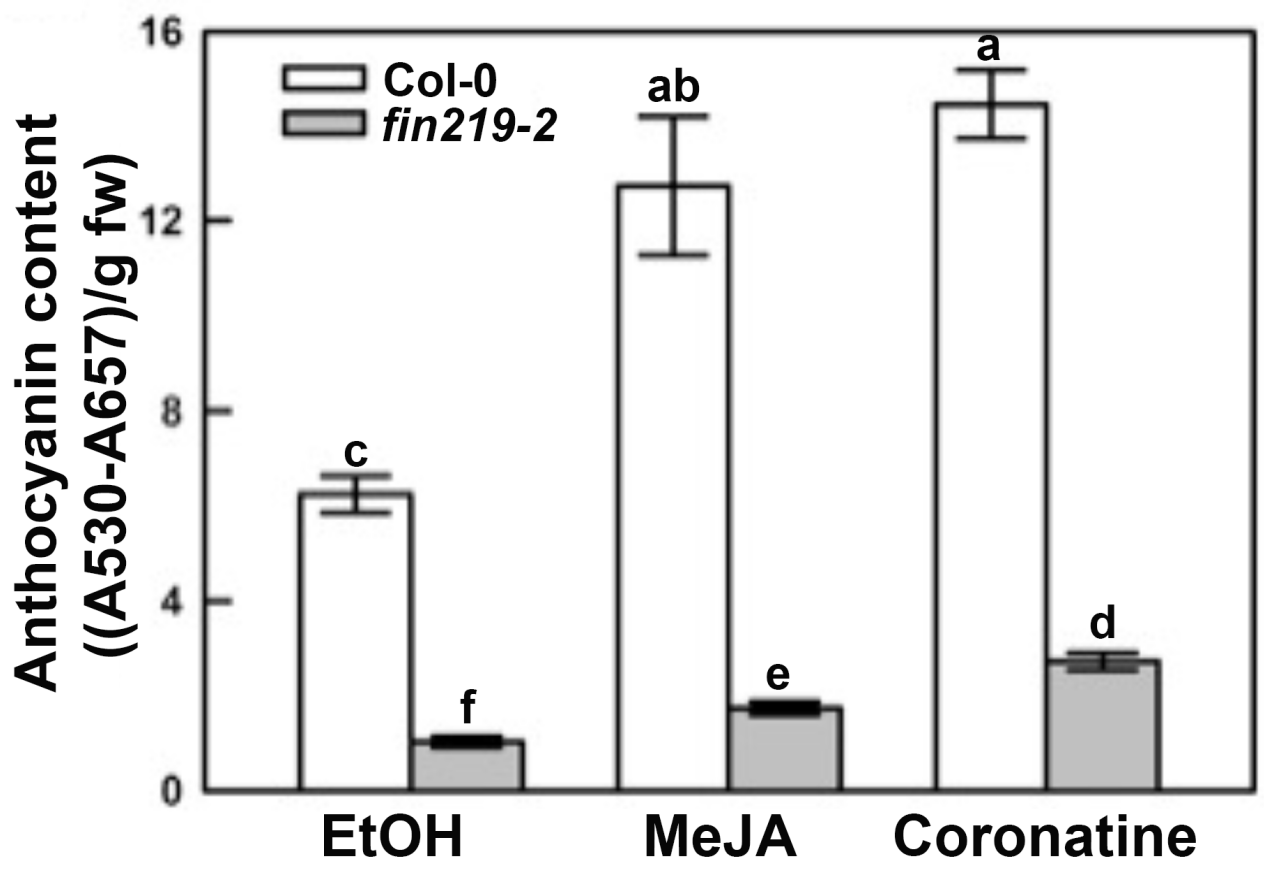

Supplemental Figure S1│ FIN219 regulates MeJA- and coronatine-induced anthocyanin accumulation in Arabidopsis seedlings. The *fin219-2* mutant accumulated less anthocyanin under MeJA and coronatine treatments. Seedlings were grown on 0.3% GM plates containing 0.1% EtOH, 50 μM MeJA, or 1 μM coronatine under cFR (1.60 μmol m^{-2} s^{-1}) for 3 days. Thirty seedlings per sample were collected for anthocyanin extraction and detection. Statistical significance was analyzed by One-way ANOVA with Fisher's LSD ($p < 0.05$) compared with their corresponding controls and denoted by different letters in the figure.

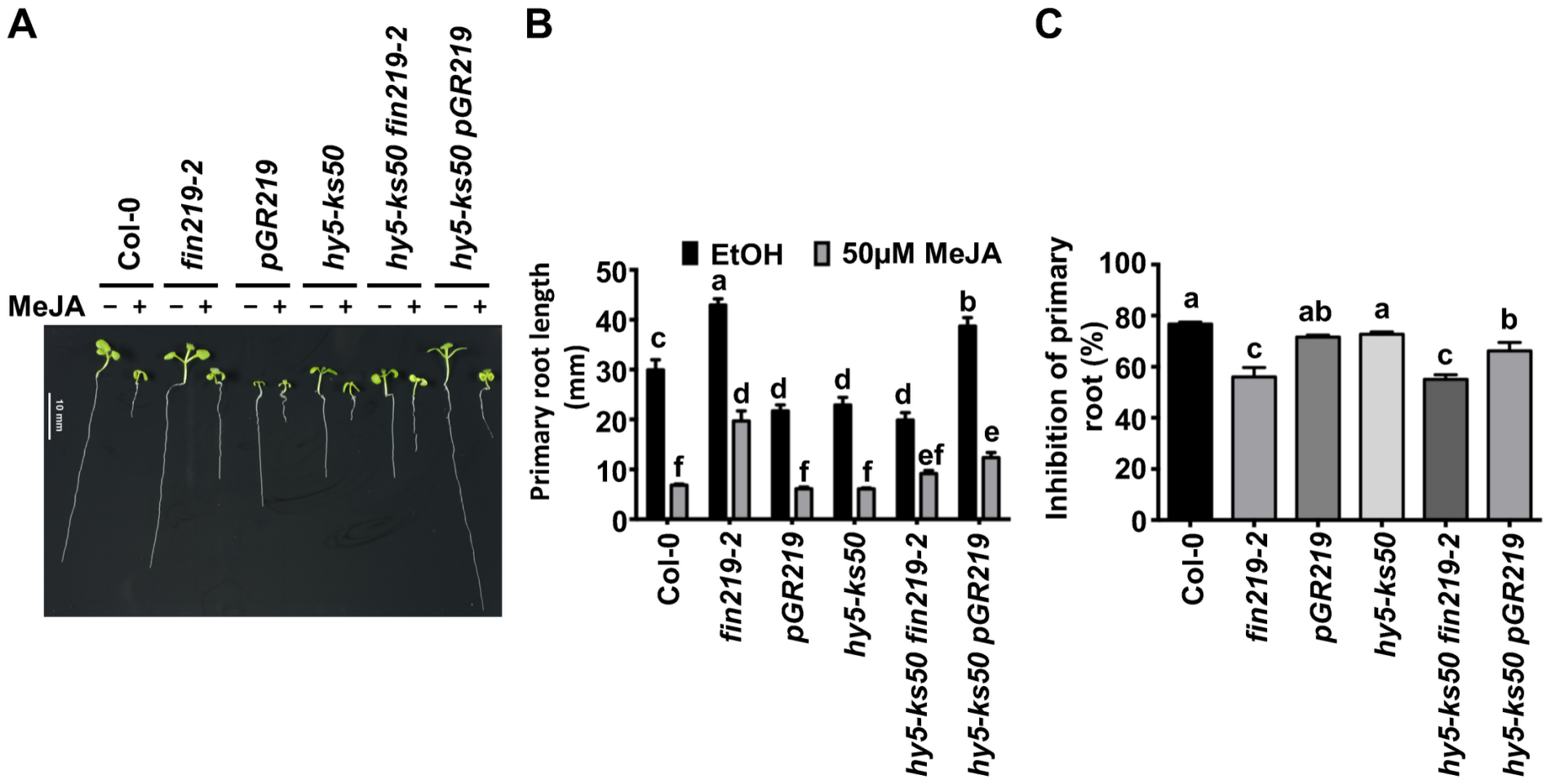

Supplemental Figure S2│ *HY5* does not affect MeJA-mediated inhibition of root elongation. (**A**) Root phenotypes of 10-day-old seedlings of Col-0, *fin219-2*, *pGR219*, *hy5-ks50*, *hy5-ks50 fin219-2*, and *hy5-ks50 pGR219* in the presence or absence of MeJA. Seedlings were grown on 0.3% GM plates containing 0.1% EtOH or 50 μM MeJA for 3 days under cWL (80 μmol m^{-2} s^{-1}). Scale bar = 10 mm. (**B**) **Quantification of primary root length.** Data are shown as mean ± SEM (n > 15). Statistical significance was analyzed by One-way ANOVA with Fisher's LSD ($p < 0.05$) compared with their corresponding controls and denoted by different letters in the figure. (**C**) Inhibition of primary root elongation by MeJA, based on the results shown in Figure S2B.

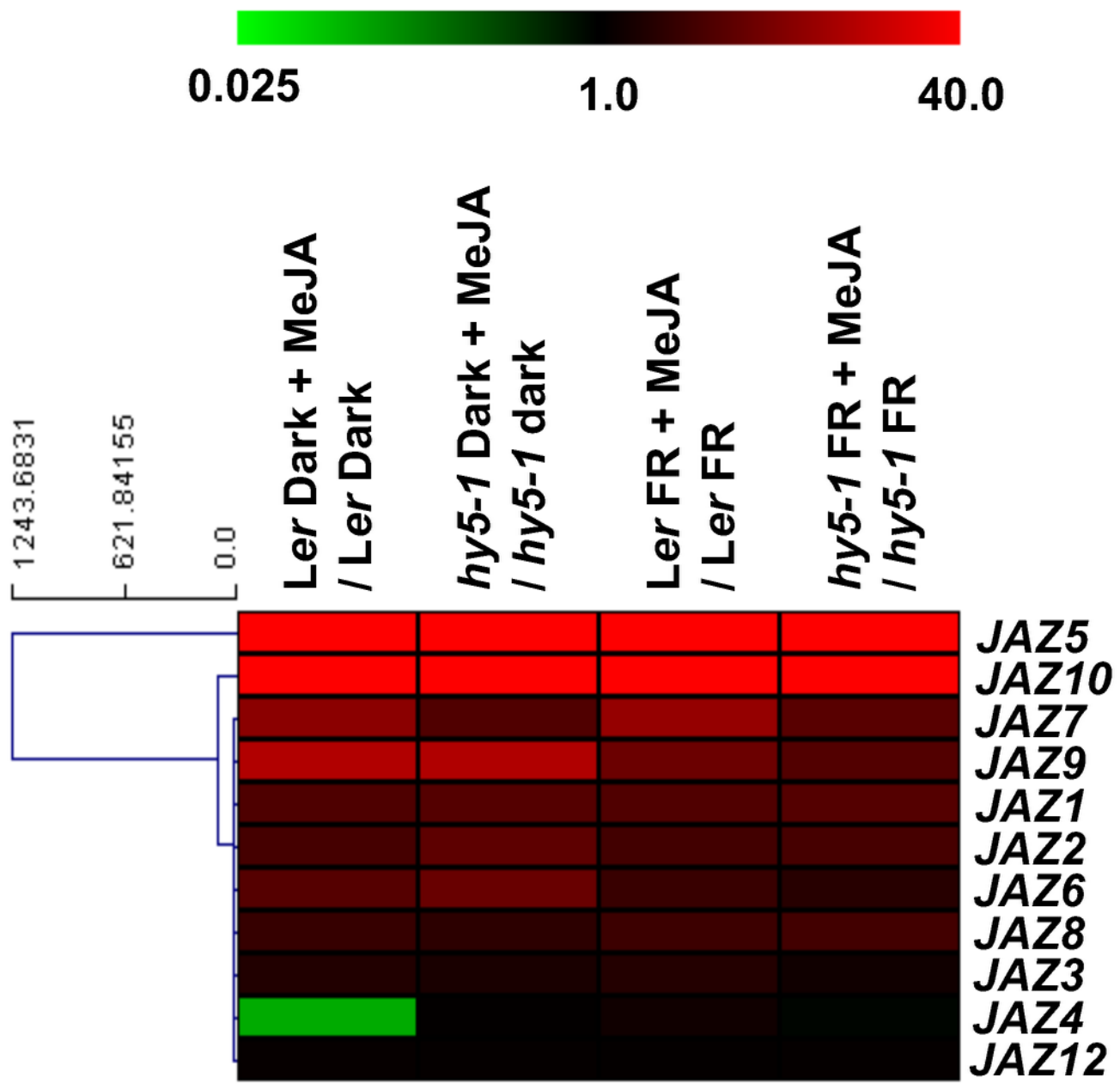

Supplemental Figure S3 | Expression profiles of *JAZ* genes identified in the microarray analysis.
The image showed hierarchical clustering based on Euclidean distances among gene fold changes.

Supplemental Table S1 | The G-box cis-elements are present in *JAZ* gene promoters.

Gene	Accession No.	G-box position (bp)
JAZ1	*At1g19180*	-283
		-1344
		**-1380**
JAZ2	*At1g74950*	-249
		-854
JAZ3	*At3g17860*	-338
		-396
		-576
JAZ4	*At1g48500*	-
JAZ5	*At1g17380*	-1025
JAZ6	*At1g72450*	-899
		-935
JAZ7	*At2g34600*	-1598
		-1699
JAZ8	*At1g30135*	-169
JAZ9	*At1g70700*	-280
		-783
JAZ10	*At5g13220*	-1483
		-1547
JAZ11	*At3g43440*	-
JAZ12	*At5g20900*	-
JAZ13	*At3g22275*	-

Supplemental Table S2 | A list of light- and JA-responsive elements present in the *JAZ1* promoter. The "Position" lane indicates the distance (base pairs) from the transcriptional start site, and "Strand" indicates the strand (+ or -) for the G-boxes located on the sense or antisense strands, respectively. The "function" descriptions are based on those from the PlantCARE database.

Site Name	Organism	Position	Strand	Sequence	Function
G-box	*Arabidopsis thaliana*	-283	+	CACGTG	cis-acting regulatory element involved in light responsiveness
G-box	*Arabidopsis thaliana*	-1380	-	CACGTG	cis-acting regulatory element involved in light responsiveness
G-box	*Arabidopsis thaliana*	-1344	+	CACGTG	cis-acting regulatory element involved in light responsiveness
G-box	*Zea mays*	-945	-	GACATGTGGT	cis-acting regulatory element involved in light responsiveness
AE-box	*Arabidopsis thaliana*	-298	-	AGAAACAA	part of a module for light response
ATCT-motif	*Arabidopsis thaliana*	-762	+	AATCTAATCT	part of a conserved DNA module involved in light responsiveness
TCT-motif	*Arabidopsis thaliana*	-740	+	TCTTAC	part of a light responsive element
TCT-motif	*Arabidopsis thaliana*	-424	-	TCTTAC	part of a light responsive element
TGACG-motif	*Hordeum vulgare*	-1089	-	TGACG	cis-acting regulatory element involved in the MeJA-responsiveness
TGACG-motif	*Hordeum vulgare*	-348	+	TGACG	cis-acting regulatory element involved in the MeJA-responsiveness
TGACG-motif	*Hordeum vulgare*	-962	-	TGACG	cis-acting regulatory element involved in the MeJA-responsiveness

Supplemental Table S3│ A list of primers used in this study.

Name	Sequence (5'-3')	Purpose
JAZ1 F	CACCCTCAAGCCGTACCAAG	For quantitative RT-PCR
JAZ1 R	GCAAGGGGATTTAGACAGGCG	For quantitative RT-PCR
JAZ2-qPCR F	GCCGAGTGTTGGGACTTCTC	For quantitative RT-PCR
JAZ2-qPCR R	GGCTGACGTGAACCTCCATT	For quantitative RT-PCR
JAZ3-qPCR F	TCGTGCCTCTGTCGATTCTTC	For quantitative RT-PCR
JAZ3-qPCR R	CCGTTTGTGCTACTTCCAGCTT	For quantitative RT-PCR
JAZ5-fw-341	AAGGCAGGGCATTCCAAAGG	For quantitative RT-PCR
JAZ5-rv-744	GCCACAGCTCTGTCTTTCCG	For quantitative RT-PCR
JAZ7-fw-208	TCCATAGCTCGTTGGACGCC	For quantitative RT-PCR
JAZ7-rv-529	CGGTGGTAAGGGGAAGTTGC	For quantitative RT-PCR
JAZ8-fw-197	CAGATGTTACCCATCTTCAGGCAA	For quantitative RT-PCR
JAZ8-rv-416	CGTCGTGAATGGTACGGTGA	For quantitative RT-PCR
JAZ9-fw-721	AGCTCTGCCACTCACACTGA	For quantitative RT-PCR
JAZ9-rv-901	TCATAAGCCTCTCTTTGCGCT	For quantitative RT-PCR
MYB28-qPCR F	TCCAACCCTACGGTCGATGA	For quantitative RT-PCR
MYB28-qPCR R	CAGAAAAGGCATTGAGCTCGAT	For quantitative RT-PCR
MYB63-qPCR F	TGAGAACAGCTCAGGCTCAAGA	For quantitative RT-PCR
MYB63-qPCR R	AGTTCGCTTTCTAGGTGCTTGAAC	For quantitative RT-PCR
AOC1-qPCR F	CGCATTCAGCTTTAGGTTTGG	For quantitative RT-PCR
AOC1-qPCR R	GTGATTCCCACGCGTTTCTT	For quantitative RT-PCR
LOX1-F1Q	CCAAGCATTACCAGCAGAAC	For quantitative RT-PCR
LOX1-R1Q	GCATACCAAACCTCAAGCC	For quantitative RT-PCR
FIN219 F	CAACTGTTTAGAVVGAGCATTCA	For quantitative RT-PCR
FIN219 R	AGTTCCTTTTGCGACAACTCTCA	For quantitative RT-PCR
CHS-qPCR F	ACGACAGGAGAAGGGTTGGA	For quantitative RT-PCR
CHS-qPCR R	GAGAGGAACGCTGTGCAAGAC	For quantitative RT-PCR
MYBL2-qPCR F	CTCTCCCCACCTTCCGGTAT	For quantitative RT-PCR
MYBL2-qPCR R	TTCGTTGTCGGTTCGTCCTG	For quantitative RT-PCR
MYB111-qPCR F	CCCTTCTCGGCAACAGATGG	For quantitative RT-PCR
MYB111-qPCR R	TGCGGCTGAGATGTGAGTTC	For quantitative RT-PCR
EcoRI-JAZ1F	GTAGAATTCATGTCAGAGTTCTATGGAATG	For cloning of JAZ1 CDS
XhoI-JAZ1R	GTACTCGAGTCATATTTCAGCTGCTAAACC	For cloning of JAZ1 CDS
EcoRI-JAZ3F	GTAGAATTCATGGAGAGAGATTTTCTCGG	For cloning of JAZ3 CDS
XhoI-JAZ3R	GTACTCGAGTTAGGTTGCAGAGCTGAGA	For cloning of JAZ3 CDS
EcoRI-JAZ4F	GTAGAATTCATGGAGAGAGATTTTCTCGG	For cloning of JAZ4 CDS
XhoI-JAZ4R	GTACTCGAGTTAGTGCAGATGATGAGCTGG	For cloning of JAZ4 CDS
EcoRI-JAZ5F	GTAGAATTCATGTCGTCGAGCAATGAAAATG	For cloning of JAZ5 CDS
XhoI-JAZ5R	GTACTCGAGCTATAGCCTTAGATCGAGATC	For cloning of JAZ5 CDS
EcoRI-JAZ6F	GTAGAATTCATGTCAACGGGACAAGCGCC	For cloning of JAZ6 CDS
XhoI-JAZ6R	GTACTCGAGCTAAAGCTTGAGTTCAAGG	For cloning of JAZ6 CDS
JAZ1-fw-D1A	GTTGCCTAAAGTTCCATTGACCTTCCCAGTAAATCTCCGGC	For deletion of JAZ1 NT domain
JAZ1-rv-D1A	AGCCGGAGATTTACTGGGAAGGTCAATGGAACTTTAGGCAACTC	For deletion of JAZ1 NT domain
JAZ1-fw-D2A	TATAAGCGGTGCCTTTGCTTTTCACAGATCTGGTAGTCTGTG	For deletion of JAZ1 ZIM domain
JAZ1-rv-D2A	CAGACTACCAGATCTGTGAAAAGCAAAGGCACCGCTAATAGC	For deletion of JAZ1 ZIM domain
JAZ1-fw-D3A	CGCTTTGGCTGGATCGCATAATTCTGTCAATGGTGTTGGGGAG	For deletion of JAZ1 Jas domain
JAZ1-rv-D3A	CCCCAACACCATTGACAGAATTATGCGATCCAGCCAAAGCGTC	For deletion of JAZ1 Jas domain
EcoRI-JAZ1-fw-58	GAATTCAAGCCTAGCTTCTCACAG	For Amplification of JAZ1 NT domain
XhoI-JAZ1-rv-156	CTCGAGTCAATCAGGCTTGCATGCCAT	For Amplification of JAZ1 NT domain
EcoRI-JAZ1-fw-379	GAATTCTTGACTATATTCTACGCCG	For Amplification of JAZ1 ZIM domain
XhoI-JAZ1-rv-459	CTCGAGTCACGCCAAGTTGATCACTTC	For Amplification of JAZ1 ZIM domain
EcoRI-JAZ1-fw-604	GAATTCCCTATTGCTAGAAGAGCTT	For Amplification of JAZ1 Jas domain
XhoI-JAZ1-rv-684	CTCGAGTCATAATTGGTATGGTGCCTTTG	For Amplification of JAZ1 Jas domain
EcoRI-HY5-fw-1	GAATTCATGCAGGAACAAGCGACTA	For amplification of HY5 NT
XhoI-HY5-rv-228	CTCGAGTCAACTTTCTCCGACAGTCG	For amplification of HY5 NT
EcoRI-HY5-fw-220	GAATTCATGGGAGAAAGTCAAAGGAAGC	For amplification of HY5 NT
XhoI-HY5-rv-507	CTCGAGTCAAAGGCTTGCATCAGC	For amplification of HY5 NT
EcoRI-HY5-fw-1	CTCGAGATGCAGGAACAAGCGACTA	For cloning of AtHY5 CDS
XhoI-HY5-rv-507	GAATTCTCAAAGGCTTGCATCAGC	For cloning of AtHY5 CDS
proJAZ1-GBOX1	ACCATGATAAAACACGTGTCATATCGAATA	For EMSA probe synthesis of G-Box1 on JAZ1 promoter
proJAZ1-GBOX2	AACAAAATAAAACACGTGTTTTTTATACCA	For EMSA probe synthesis of G-Box2 on JAZ1 promoter
proJAZ1-GBOX3	GGTACAGACAACGTGCACTTGCCAACCTTC	For EMSA probe synthesis of G-Box3 on JAZ1 promoter
